# chewBBACA 3: lowering the barrier for scalable and detailed whole- and core-genome multilocus sequence typing

**DOI:** 10.1186/s13073-026-01625-x

**Published:** 2026-03-24

**Authors:** Rafael Mamede, Pedro Vila-Cerqueira, João André Carriço, Mario Ramirez

**Affiliations:** 1 Instituto de Microbiologia, Faculdade de Medicina, Universidade de Lisboa, Av Prof Egas Moniz, Lisboa, 1649-028 Portugal; 2 Gulbenkian Institute for Molecular Medicine, Oeiras, Portugal; 3 Present address: bioMérieux Portugal, Linda-a-Velha, 2795-197 Portugal

**Keywords:** Bacterial genomics, Genomic epidemiology, Pathogen surveillance, Outbreak detection, Public health, Bacterial typing, wg/cgMLST

## Abstract

**Background:**

The wide adoption of whole genome sequencing has enabled the implementation of genomics-based systems, which provide unparalleled resolution for the surveillance and outbreak investigation of bacterial pathogens. To fully exploit the wealth and complexity of genomics data, bioinformatics methods need to be highly scalable, provide accurate and extensive data for potential downstream analyses, as well as analytic capabilities. Here, we present chewBBACA 3, a suite of modules for scalable and comprehensive bacterial whole- and core-genome multilocus sequence typing (wg/cgMLST) with built-in features to create new schemas, evaluate loci diversity and strain similarity.

**Results:**

chewBBACA 3 enables faster and more accurate schema creation and allele calling by complementing an alignment-based approach with alignment-free methods, including hash-based comparisons and minimizer-based clustering. Schema creation is up to 55-fold faster and identifies up to 10% more loci than its predecessor, chewBBACA 2. Furthermore, chewBBACA 3 can quickly adapt or import schemas available on external wg/cgMLST platforms or Chewie-NS, promoting interoperability. The efficiency of allele calling allows processing larger genome collections, from thousands to tens of thousands of genomes, at the whole- and core-genome levels without requiring high computational resources and being up to 52-fold faster than similar tools. chewBBACA 3’s enhanced sensitivity allows it to identify and classify more schema loci and coding sequences than the compared methods, resulting in higher resolution for strain comparison. Moreover, the allelic profiles, classification statistics and associated sequence data produced by chewBBACA 3 can be the basis for detailed analyses that provide added value in surveillance and outbreak investigation settings. New modules leverage the potential of the schema and allele call results data to create interactive reports that enable an intuitive and in-depth analysis of allele diversity in loci of interest and allow assessing strain similarity based on loci presence, allelic distances and phylogenetic analysis.

**Conclusions:**

chewBBACA 3 provides functionalities for complete wg/cgMLST analysis at scale, lowering the barrier for the use of wg/cgMLST and offering extensive results and analytic capabilities for streamlined, comprehensive, and local analyses. chewBBACA 3 is freely available at https://github.com/B-UMMI/chewBBACA.

**Supplementary Information:**

The online version contains supplementary material available at 10.1186/s13073-026-01625-x.

## Background

The burden of bacterial infections constitutes a major challenge to public health systems worldwide [1, 2]. The advances in sequencing technologies have enabled public health institutions to support and gradually transition to whole genome sequencing (WGS), increasing surveillance capacity and the effectiveness of outbreak investigations. WGS offers high-resolution discrimination of closely related bacterial strains and enables the identification of pathogens’ relevant features in a timely and accurate manner, aiding in reaching an informed decision for effective disease prevention and control [3–6]. The widespread use of WGS, as well as the adherence to FAIR principles, encouraged the development of efficient bioinformatics methods to perform the in silico equivalent of commonly applied bacterial typing techniques, such as multilocus sequence typing (MLST) [7, 8]. It also allowed transitioning to methods with enhanced resolution that leverage the full genomic content to identify relevant features and provide a more accurate measure of strain similarity [9]. These methods are diverse but generally adopt one of three fundamental approaches: (i) determining Single-Nucleotide Variants (SNVs) relative to a reference genome, (ii) measuring sequence similarity based on short subsequences of length *k*, known as *k*-mers [10], and (iii) comparing the strains’ gene content, referred to as gene-by-gene methods (GbG) [9].

SNV approaches detect differences at the single nucleotide level by mapping sequencing reads against a closely related reference strain. The precision level of this approach enables the identification of point mutations or more complex variants that can be determinants of phenotypic characteristics of interest, such as increased virulence and antimicrobial resistance. The choice of the reference genome is crucial as the quality and relatedness of the reference genome to the strains of interest can greatly influence the number of shared positions compared and, therefore, the extent of the variability detected [11, 12]. *k*-mer-based tools split genomic sequences into *k*-mers and compare the resulting *k*-mer sets to estimate strain similarity or identify regions of interest. These approaches can estimate similarity without needing a reference genome and are potentially faster and more computationally efficient than SNV or GbG approaches. The efficiency of these approaches depends on the sampling method used to select *k*-mers, which should be fine-tuned to achieve a good balance between efficiency and accuracy for the desired application [13, 14]. With the wide adoption of WGS, GbG approaches have transitioned from classical MLST to whole-genome and core-genome MLST (wg/cgMLST). wg/cgMLST enables the creation of schemas encompassing the variability of hundreds to thousands of loci for a species of interest to accurately determine the loci and alleles present in strains of interest. Creating and maintaining wg/cgMLST schemas to accurately capture a species’ diversity can be a laborious process [15, 16]. As with SNV approaches, knowing the alleles present at a given locus can be linked to phenotypic properties such as virulence or antimicrobial resistance.

It has been shown that applying any of these approaches can generate results suitable for accurate strain similarity estimation and phylogenetic analyses in surveillance and outbreak scenarios [9, 17]. Nevertheless, wg/cgMLST has been more frequently integrated into surveillance and outbreak detection systems, partly due to constituting an expansion of classical MLST, which conceptually and technically allows for a more straightforward implementation, especially in constantly growing datasets such as the ones used in long-term epidemiological surveillance. The capacity to update wg/cgMLST schemas with new alleles increases the diversity captured by and, consequently, the resolution of wg/cgMLST analyses. Moreover, wg/cgMLST allows establishing allelic nomenclatures for standardised comparisons. Existing solutions for wg/cgMLST analysis can vary greatly in the degree of data centralisation, analytical capabilities, and license type [18–20]. To continue to promote the adoption of wg/cgMLST, improvements should focus on interoperability to facilitate comparison of results, scalability to meet growing data processing demands, and easily performed comprehensive local analyses to offer powerful analytic capabilities to end users while complying with strict data privacy laws.

To provide a solution for scalable, detailed, and local wg/cgMLST, we developed chewBBACA 3, which vastly improves and extends the functionalities of chewBBACA 2 [20, 21], a widely used tool for wg/cgMLST which has been integrated into public health workflows such as EFSA’s One Health WGS system, used for rapid detection of multi-country foodborne outbreaks in collaboration with the European Centre for Disease Prevention and Control (ECDC) [6].

## Implementation

### Overview

**Fig. 1:**
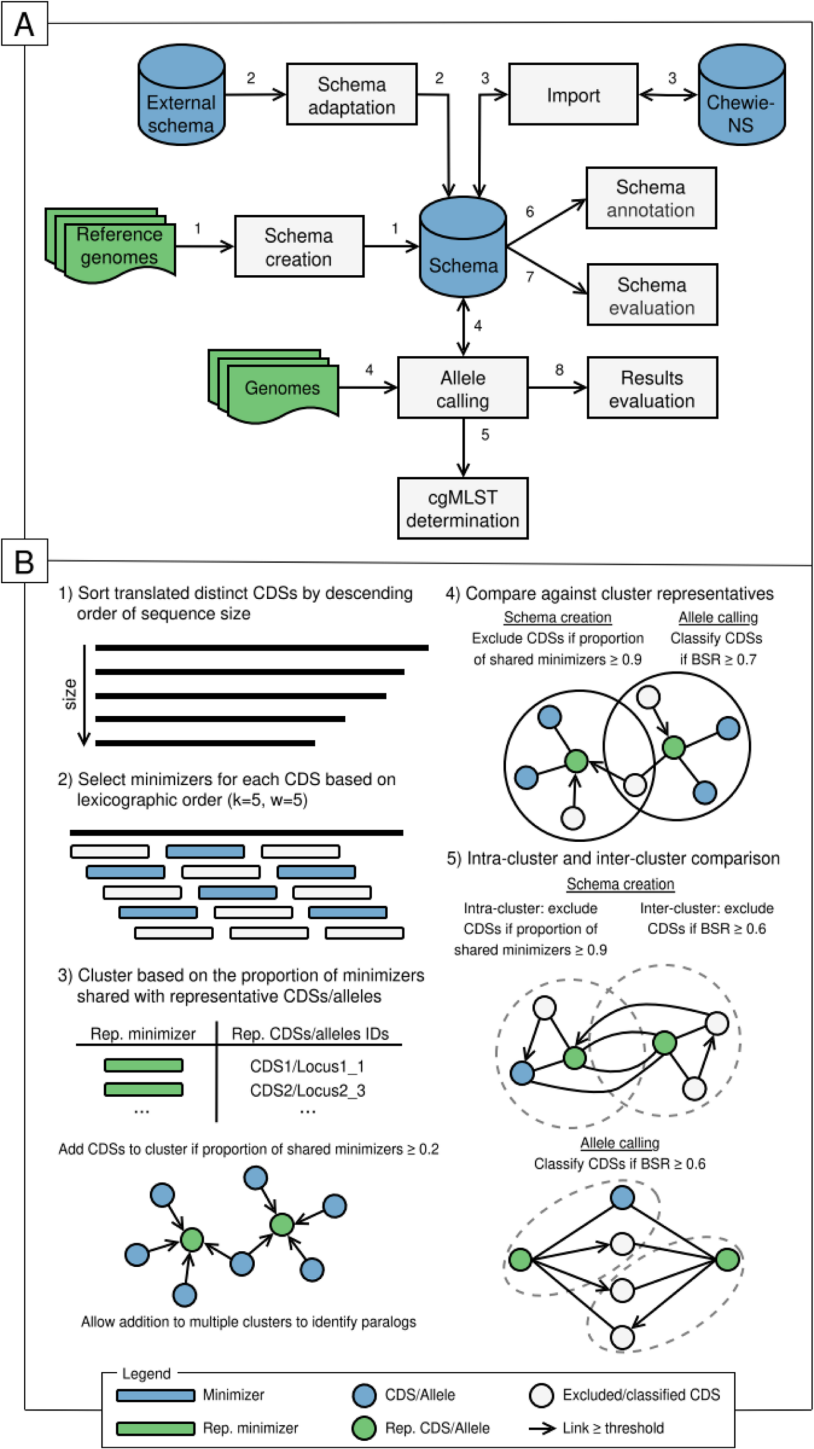
Overview of chewBBACA 3’s processes and minimizer-based clustering used by the *CreateSchema* and *AlleleCall* modules. (**A**) chewBBACA 3 includes modules for schema setup (steps labeled with 1, 2 and 3), allele calling (steps labeled with 4), core genome determination (steps labeled with 5), schema annotation (steps labeled with 6), schema evaluation (steps labeled with 7), and results evaluation (steps labeled with 8). Blue cylinder icons represent schemas, with the central cylinder icon representing a schema created or adapted for usage with chewBBACA 3. Green document icons represent input FASTA files. Grey rectangle icons represent analysis processes available in chewBBACA 3. (**B**) Minimizer-based clustering and classification steps implemented in the *CreateSchema* and *AlleleCall* modules. (Step 1) The distinct translated CDSs not classified through exact matching at the DNA and protein levels are sorted based on decreasing size. (Step 2) Minimizers are selected from the set of 5-mers for each CDS based on lexicographic order and a window size of 5. (Step 3) The set of minimizers selected for each CDS is compared against the minimizers of CDSs selected as cluster representatives (*CreateSchema*) or the schema loci representative alleles (*AlleleCall*) to cluster CDSs based on a proportion of shared minimizers ≥ 0.2. (Step 4) The CDSs that share a proportion of minimizers ≥ 0.9 (*CreateSchema*) or a BSR ≥ 0.7 (*AlleleCall*) with the cluster representative are excluded from the analysis (*CreateSchema*) or classified (*AlleleCall*). (Step 5) Non-representative CDSs from the same cluster are compared to exclude smaller CDSs that share a proportion of minimizers ≥ 0.9 with larger CDSs (*CreateSchema*). Representative CDSs or alleles are aligned against all CDSs to exclude (*CreateSchema*) or classify (*AlleleCall*) CDSs based on a default BSR value of 0.6

chewBBACA 3 is a complete reimplementation of its predecessor, chewBBACA 2 [20], which was already an upgraded version of chewBBACA’s first published version [21]. chewBBACA 3 provides a modular approach for complete wg/cgMLST analysis (Fig. 1A), offering efficient and accurate schema creation and allele calling and allowing for a comprehensive evaluation of schemas and results through interactive reports. chewBBACA 3 allows setting up schemas for wg/cgMLST analysis from large collections of genome assemblies or coding DNA sequences (CDSs) in FASTA format or by adapting existing schemas from external platforms [18, 19, 22]. Additionally, the integration with Chewie-NS, a web server used to store and manage wg/cgMLST schemas and a common allele nomenclature, which was previously described together with chewBBACA 2 [20], allows easily importing ready-to-use schemas to obtain comparable interlaboratory results based on a common allelic nomenclature. To determine the allelic profiles of strains of interest, chewBBACA 3 identifies and clusters the distinct CDSs predicted from the strains’ genomes, significantly reducing the number of comparisons against the schema loci in contrast to the sequential strain processing used by chewBBACA 2. This translates into faster and more efficient allele calling and facilitates data aggregation to create output files with more detailed results. Allele calling identifies and adds new alleles to schemas, ensuring that they are gradually updated to produce accurate and comparable results over time. Similarly to chewBBACA 2, new alleles are inferred based on the BLAST Score Ratio (BSR) [23] computed from BLASTp alignments [24], complying with minimum sequence length and allele size variation thresholds [21]. Adjustments to these parameters allow chewBBACA 3 to classify more CDSs and capture loci diversity more accurately. chewBBACA 3 increases the granularity of the results by expanding the set of special classifications assigned when the presence of a locus cannot be inferred confidently, such as when a CDS matching a schema locus is outside the user-specified locus size variation interval or if multiple CDSs from a genome match the same schema locus (Additional file 1: Fig. S1-S4). These special classifications aid in identifying spurious alleles resulting from low-quality data, pseudogenes, and paralogous loci. The set of core loci can be determined based on the allele calling results for any locus presence threshold, and the resulting list of core loci can be used to perform allele calling at the core genome level. Schema loci can be annotated by searching for exact matches through UniProt’s SPARQL endpoint, which retrieves and selects annotations based on all UniProt entries and was already an option in chewBBACA 2, and now also by downloading UniProt’s reference proteomes for specific *taxa* and aligning the schema’s alleles against the proteome entries to retrieve annotations based on entries selected to represent the diversity of the specified taxa [25]. New schema and results evaluation modules leverage the power of the React JavaScript library [26] to build interactive reports that enable local and comprehensive analyses of the diversity of loci contained in schemas and aid in identifying closely related strains for more effective surveillance and outbreak assessment.

### Core modules

While all the modules in chewBBACA 2 were reimplemented to increase the scalability and comprehensiveness of the results generated by chewBBACA 3, module development concentrated primarily on the *CreateSchema* and *AlleleCall* modules (Additional file 1: Fig. S5 and S6), which handle schema creation and allele calling, respectively. Gene prediction was optimised in both modules using Pyrodigal [27, 28], a Python module that provides bindings to Prodigal for seamless integration and offers several advantages, such as faster gene prediction and greater control over gene prediction parameters and results. Additionally, a new feature was added allowing both modules to accept FASTA files with CDSs, enabling users to leverage the vast CDS data available in public databases or provide CDSs predicted by other gene prediction tools if preferred by the user, such as GeneMarkS-2 or Balrog [29, 30]. Following the gene prediction step, CDS deduplication is performed at both DNA and protein levels (i.e. identify different DNA sequences encoding the same protein) to identify the set of distinct CDSs. The distinct translated CDSs are clustered based on the proportion of shared minimizers (≥ 0.2) with representative alleles [31–33] (Fig. 1B:1–3). The minimizer parameters (k = 5, w = 5, lexicographic order) were chosen to select sets of *k*-mers that cover most sequence positions at least once while also keeping memory usage low [34]. For schema creation, the CDSs sharing a high proportion of minimizers (≥ 0.9) with the cluster representative or larger CDSs are considered alleles of the same locus and are excluded (Fig. 1B:4). The clustering results are complemented by intracluster and intercluster alignment with BLASTp to exclude CDSs based on the BSR threshold and select the final set of CDSs used to create a schema seed with one representative allele for each locus (Fig. 1B:5). This schema seed will be used by the *AlleleCall* module to determine the allelic profiles of strains of interest and add new alleles identified in the classified strains to the schema. Another new feature implemented is that each distinct CDS is hashed, mapped to the compressed list of genomes that contain it (Additional file 1: Fig. S7) and compared against the hashed schema alleles. This allows keeping the information about the CDSs identified in all strains in memory, enabling fast exact matching and classification of all genomes containing a CDS based on a single match. Clustering and intracluster alignment with BLASTp allow comparison of the remaining unclassified CDSs against the schema’s representative alleles to find and classify inexact matches (Fig. 1B:1–4 ). A final step aligns the schema’s representative alleles against the remaining unclassified CDSs to find more divergent alleles and select new representative alleles (Fig. 1B:5). The matches found throughout the process are evaluated at the end of the process to assign the final classifications, create the allelic profiles, and update the schema with novel alleles. Both core modules create several output files with detailed schema and results data that support the analyses performed by other modules and can serve as the basis for custom analyses that seek to answer relevant questions at the strain or population-wide level.

A more detailed description of the implementation and functionalities included in each module is available in the supplementary material (Additional file 1) and in chewBBACA’s online documentation [35].

### Download and selection of complete and draft genome assemblies

Complete and draft genome assemblies annotated as *Streptococcus pyogenes*, *Listeria monocytogenes* and *Salmonella enterica* were downloaded with the NCBI Datasets command-line tools v16.12.0 [36] on September 9, 2023. The complete genomes were downloaded from the NCBI RefSeq database [37] using the *–assembly-source “RefSeq”* and *–assembly-level complete* options. The draft genome assemblies were downloaded from the NCBI GenBank database [37] using the *–assembly-source “GenBank”* option. The *–exclude-atypical* and *–mag exclude* options were used in both cases. The number of draft genome assemblies for *S. pyogenes* available from GenBank was insufficient to create the complete dataset (n = 16384) for the benchmark. Due to that, draft genome assemblies annotated as *Streptococcus pyogenes* were also downloaded from a collection of 661 K genomes available on the European Nucleotide Archive (ENA) [38]. MLST v2.23.0 [18, 39] was used to determine the Sequence Type (ST) for all assemblies. Assemblies without a known ST or assigned an ST from a different species, indicating possible misannotation, were excluded. A custom Python script was also used to filter out assemblies based on a maximum number of contigs of 100, a maximum number of ambiguous bases of 1000, and a minimum and maximum genome size. The minimum and maximum genome size values were defined based on the *min_ungapped_length* and *max_ungapped_length* values in the “species_genome_size.txt” file available on NCBI’s FTP on September 9, 2023 (https://ftp.ncbi.nlm.nih.gov/genomes/ASSEMBLY_REPORTS/*)* [37].

### Dataset creation

The selected draft genome assemblies were subsampled to create datasets to evaluate the performance of chewBBACA 3, chewBBACA 2 and pyMLST. The pairwise average nucleotide identity (ANI) distances for each species’ selected draft genomes were computed with Skani v0.2.1 [40]. To factor in the aligned genome fraction, weighted ANI values were computed by multiplying the ANI values by the mean of the query and reference aligned fractions. The weighted ANI values were ordered to select a set of 16,384 genomes that maximized the average pairwise distance. Smaller datasets were created by randomly sampling this dataset, starting by selecting 1 genome and doubling the dataset size until reaching a dataset size of 8,192. Five replicates were created for each dataset size. The complete datasets with 16,384 genomes were compressed with AGC v3.0 [41] to allow efficient storage and fast genome retrieval based on lists of genome identifiers.

### Creation of wg/cgMLST schemas

A total of 260, 309 and 1,326 complete genomes for *Streptococcus pyogenes*, *Listeria monocytogenes* and *Salmonella enterica*, respectively, were selected for schema creation. wgMLST schema seeds were created with the *CreateSchema* module available in chewBBACA v3.3.6 and compared against the schema seeds created by the previous *CreateSchema* implementation, available in chewBBACA v2.6.0 [21]. The schema creation processes used a minimum sequence length value of 0 (*--l 0*) and the Prodigal [28] training files bundled with chewBBACA. The schema seeds created by both versions were compared based on a BSR ≥ 0.6 and a proportion of shared minimizers ≥ 0.9 to determine sets of loci shared by the schema seeds created with both versions. Schema seeds created with chewBBACA v3.3.0 were populated with the alleles identified in the complete genomes through allele calling. The results of the allele calling were used to determine the set of core loci with the *ExtractCgMLST* module based on a loci presence threshold of 1 (--t 1) and create the cgMLST schemas used to evaluate the allele calling performance. The cgMLST schemas were adapted with the *PrepExternalSchema* module implemented in chewBBACA v2.8.5 to create the cgMLST schemas for that version. To create equivalent databases for pyMLST [42], multi-FASTA files with the first representative allele for each locus in the cgMLST schemas were passed to the *wgMLST create* command. The *wgMLST add* command was used to add each complete genome to the pyMLST databases.

### External schema adaptation

The cgMLST schemas for *S. pyogenes*, *L. monocytogenes* and *S. enterica* available on the cgMLST.org server [22] were downloaded on July 4, 2024. These schemas were adapted with the *PrepExternalSchema* module available in chewBBACA v3.3.6 and compared against the schemas adapted with the previous *PrepExternalSchema* implementation, available in chewBBACA v2.0.17.2. The representativeness of the set of representative alleles selected by the *PrepExternalSchema* module was measured by aligning the representative alleles selected for each locus against all valid locus alleles based on a BSR ≥ 0.6.

### Evaluation of the allele calling results

The cgMLST schemas and datasets containing between 1 and 16,384 draft genome assemblies were used to evaluate the allele calling performance of chewBBACA v3.3.3, chewBBACA v2.8.5 and pyMLST v2.1.5. The number of distinct CDSs per dataset was computed based on the CDSs predicted by Pyrodigal v3.0.0. Runtime, peak memory usage, and the comprehensiveness of the allele calling were evaluated for all datasets. The allelic profiles for the strains classified by pyMLST were extracted from the databases with the *wgMLST mlst* command and converted to the format used by chewBBACA with a custom script. The allelic profiles were masked to remove the *INF-* prefix from inferred alleles and to substitute all special classifications or missing values by 0. The core loci were defined with the *ExtractCgMLST* module based on the complete datasets’ results and a loci presence threshold of 0.95. Loci below this threshold were considered to be part of the accessory genome. The pairwise Jaccard and allelic distances were computed with a custom script based on the masked allelic profiles. The proportion of classified CDSs and identified loci are based on the total number of CDSs predicted by Pyrodigal and on the total number of loci in each schema, respectively.

### Download and analysis of *S. pyogenes emm1* strains

The genome assemblies and metadata for the *S. pyogenes* strains belonging to each lineage were recovered from previous studies [15, 43, 44]. The schema loci containing the lineage-defining SNPs were identified using BLASTp to align the translated CDSs from the MGAS5005 reference genome [45], with RefSeq accession number GCF_000011765.3, against the translated schema alleles.

### Runtime and peak memory usage measurement

Runtime and peak memory usage were measured with the GNU time command on a desktop computer with an Intel^®^ Core™ i7-4790 CPU, 32GB 1600 MT/s RAM, and a 1 TB Samsung SSD 870 QVO. Any analysis that evaluated runtime and peak memory usage used 6 CPU cores to run chewBBACA 3 and chewBBACA 2 and 1 CPU core for pyMLST because the latter cannot use multiple cores.

## Results and discussion

### Fast wg/cgMLST schema creation or retrieval from multiple sources

chewBBACA 3 offers three options for setting up a schema for wg/cgMLST analysis.

The first option is creating a new schema seed by selecting loci from a set of complete or draft genome assemblies with the *CreateSchema* module (Additional file 1: Fig. S5). To evaluate the performance of the *CreateSchema* module, we created schema seeds with chewBBACA 3 and chewBBACA 2 based on the complete genome assemblies available on the NCBI RefSeq database [37] for three bacterial species: *Streptococcus pyogenes* (*n* = 260), *Listeria monocytogenes* (*n* = 309), and *Salmonella enterica* (*n* = 1,326). Schema seed creation was 25- to 55-fold faster with chewBBACA 3 than with chewBBACA 2, with similar memory usage (Additional file 2: Table S1). A comparison of the schema seeds generated with both versions revealed that the schema seeds created by chewBBACA 3 contained 98% of the loci identified by chewBBACA 2 (Additional file 2: Table S2). Moreover, chewBBACA 3 identified 6% to 10% more loci than chewBBACA 2, primarily due to a more accurate identification of smaller loci. Ideally, target loci should be defined based on a set of high-quality genome assemblies to avoid the inclusion of spurious loci in the schema seed. Nonetheless, schema seed creation with chewBBACA 3 will remain efficient even when using larger genome collections, possibly including draft genomes, to adequately capture a species diversity.

A second option is to adapt schemas from external platforms with the *PrepExternalSchema* module (Additional file 1: Fig. S8). This module filters out incomplete alleles (i.e. alleles that contain ambiguous bases or do not correspond to valid CDSs, such as having no start/stop codon) and selects representative alleles based on a BSR threshold to create a schema structure compatible with chewBBACA. Schema adaptation promotes the usage of schemas previously made available and adopted by the community, contributing to the integration and interoperability with other platforms. chewBBACA 3 is over three orders of magnitude faster than chewBBACA 2 when adapting the cgMLST schemas for *S. pyogenes*, *L. monocytogenes*, and *S. enterica* available on the cgMLST.org server [22], adapting any of the schemas in under five minutes (Additional file 2: Table S3). Furthermore, in contrast to chewBBACA 2, chewBBACA 3 now ensures that the selected representative alleles fully capture the diversity of each locus based on the specified BSR threshold (Additional file 2: Table S4). chewBBACA 3 also provides options to filter out alleles based on a user-defined minimum sequence size and locus size variation thresholds (e.g. for a size variation threshold of 0.2, any potential alleles with a sequence size below or above 20% of the mode of the allele size are excluded during schema adaptation) and outputs detailed information about the changes made while adapting a schema to inform the user of the changes introduced to the existing schema.

Lastly, the third option is the *DownloadSchema* module (Additional file 1: Fig. S9), one of the modules (Additional file 1: Fig. S9-S11) developed to integrate with Chewie-NS [20], allowing users to import ready-to-use schemas from Chewie-NS instances. This option offers the advantage of enabling local and private analysis based on a common allelic nomenclature to facilitate the comparison of results. Schemas downloaded from Chewie-NS can be kept up-to-date by synchronizing with the remote versions to receive the latest allele data submitted by other users and, if desired, contribute novel alleles identified locally.

### Scalable and efficient allele calling

**Fig. 2:**
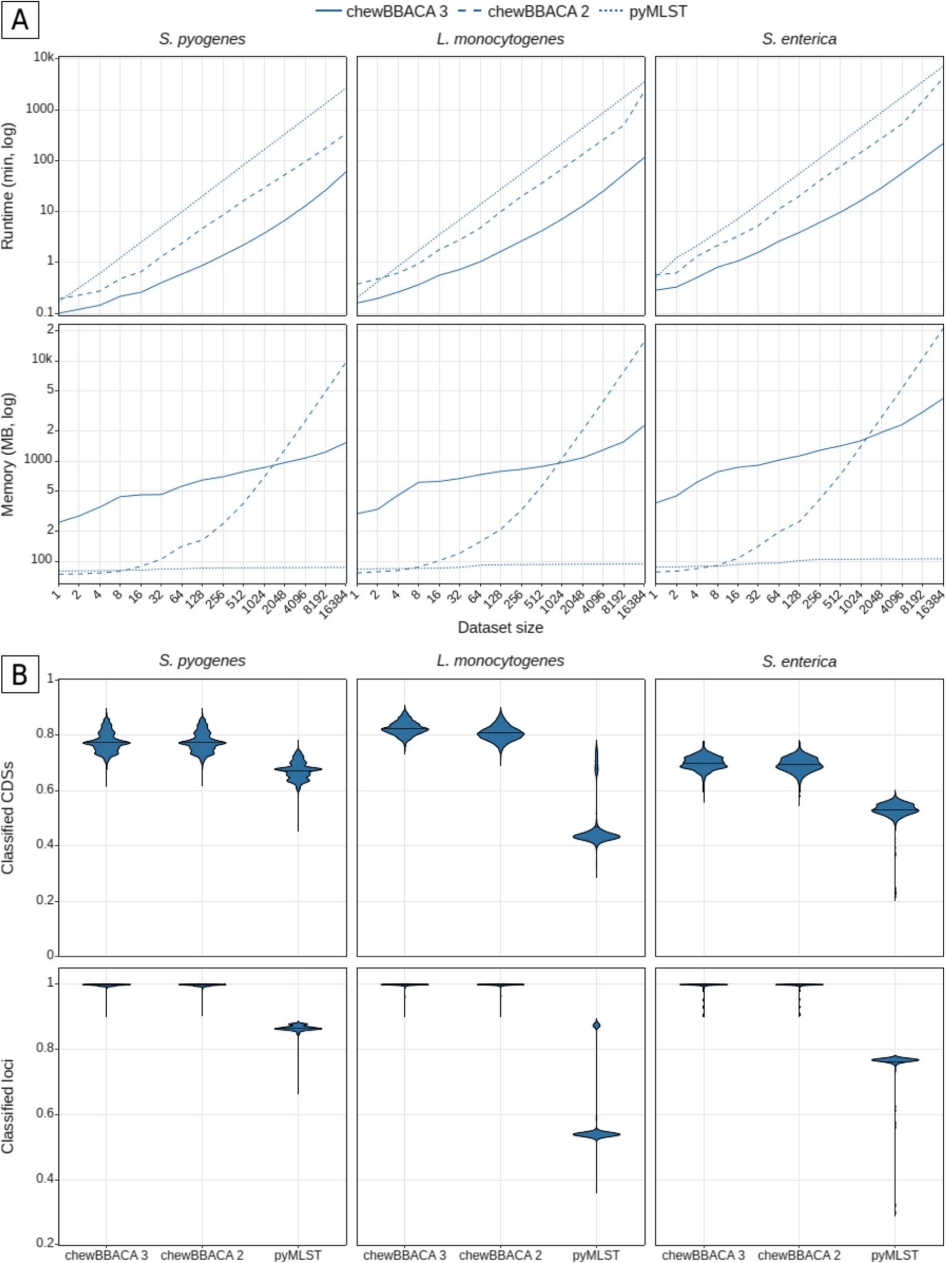
Performance comparison of chewBBACA 3, chewBBACA 2 and pyMLST. (**A**) Runtime and peak memory usage comparison for the allele calling of datasets with a varying number of genomes (from 1 to 16,384) for three bacterial species: *Streptococcus pyogenes*, *Listeria monocytogenes*, and *Salmonella enterica*. The benchmark was performed with five replicates per dataset size, except for the complete dataset (*n* = 16384). The values shown are the mean of the replicate values for each dataset. Runtime was measured as the elapsed real time in minutes (logarithmic scale). Peak memory usage was measured as the maximum resident set size in MB (logarithmic scale). (**B**) Proportion of strain CDSs and schema loci classified for the complete datasets (*n* = 16384). The proportion of classified CDSs corresponds to the number of CDSs classified by each tool divided by the total number of CDSs predicted for each strain by Pyrodigal. The proportion of classified loci corresponds to the number of schema loci identified by each tool divided by the total number of schema loci

We performed allele calling with the schema seeds created with chewBBACA 3 in the previous section and the complete genomes for each species to add new alleles to the schemas and determine the set of core loci with the *ExtractCgMLST* module (Additional file 1: Fig. S12). The lists of core loci (present in 100% of the genomes) were used to measure performance at the cgMLST level for datasets including between 1 and 16,384 draft genome assemblies and compared against the results obtained with chewBBACA 2 and pyMLST [42] for equivalent schemas and databases (Additional file 2: Table S5). chewBBACA 3 processed all datasets faster than chewBBACA 2 and pyMLST. On average, chewBBACA 3 was 1.9- to 20.3-fold and 1.3- to 51.9-fold faster than chewBBACA 2 and pyMLST, respectively (Fig. 2A and Additional file 2: Table S6). The difference increased with dataset size, largely due to the increased redundancy (same sequence found in different genomes) of the set of CDSs extracted from the genomes (Additional file 2: Table S7). For example, only 1.5% to 2.8% of the total CDSs identified in the complete datasets (*n* = 16,384) were distinct. By identifying the set of distinct CDSs before trying to match them against the schema loci, chewBBACA 3 avoids the repeated evaluation of identical CDSs identified in multiple genomes. Additionally, the new minimizer-based clustering matches the remaining CDSs to the most similar schema loci, reducing comparisons between dissimilar sequences. These two steps contribute the most to the increased speed compared to chewBBACA 2 and pyMLST, which process each genome separately and additionally do not take advantage of multiprocessing settings as efficiently as chewBBACA 3. Regarding peak memory usage (Fig. 2A and Additional file 2: Table S8), chewBBACA 3 used, on average, 8.6- to 1.1-fold more memory than chewBBACA 2 for datasets with up to 1,024 strains. The inverse was observed for larger datasets, with chewBBACA 2 using 1.1- to 6.9-fold more memory than chewBBACA 3. Compared to pyMLST, chewBBACA 3 used 3.1- to 40-fold more memory. pyMLST maintains low memory usage irrespective of dataset size but is single-threaded and only supports the addition of one strain per command, which limits its scalability. chewBBACA 3 enables considerably faster analyses while keeping memory usage in check to allow large-scale analysis without needing high-performance computing infrastructures. While comparing results using the entire wgMLST schema would further highlight chewBBACA 3’s efficiency and accuracy, time and memory constraints related to running chewBBACA 2 and pyMLST under the same conditions invalidated such comparison.

The thoroughness of the allele calling in chewBBACA 3 can be controlled through four execution modes (Additional file 1: Fig. S6). Mode 1 identifies exact matches at the DNA level between the genomes’ CDSs and the schema alleles. Mode 2 adds exact matching at the protein level, enabling the identification of novel alleles with synonymous substitutions. Mode 3 proceeds to clustering and intracluster alignment to identify similar alleles based on the BSR threshold. Mode 4, the default, runs the complete process to classify as many CDSs as possible and potentially selects new representative alleles, preparing the schema to better identify future novel alleles. Modes 1 and 2 offer up to a 4.7-fold speedup over the default mode (Additional file 1: Fig. S13 and Additional file 2: Tables S9 and S10), but this speedup is most noticeable for smaller datasets, gradually decreasing as dataset size increases, possibly due to a larger overhead of the gene prediction step. Additionally, mode 1 cannot identify new alleles and mode 2 is limited to the identification of new alleles with synonymous substitutions. This makes them appropriate for applications where a less accurate but much faster strain discrimination is sufficient, or for faster allele calling of small sample batches with schemas that already capture most of a species’ diversity for the set of loci that make up those schemas, as is the case for many publicly available cgMLST schemas. Mode 3 provides similar accuracy to the default mode in less time, with a more significant reduction in runtime for larger schemas and more diverse datasets. Mode 4 offers greater sensitivity to identify the most divergent alleles and select new representative alleles to add to schemas, which is essential to increase the diversity captured by a schema, especially in the initial phase of schema development. For schemas that already include representative alleles that capture a species diversity, Mode 3 and Mode 4 may only differ in the number of special classifications attributed, with Mode 4 identifying more.

chewBBACA 2 added new alleles to schemas automatically, not providing any option for users to prevent the allele call process from changing an existing schema. chewBBACA 3 includes the *--no-inferred* option to control this behaviour. This option can be helpful in several scenarios, including: updating schemas only periodically, in applications where frequent schema updates can compromise the reproducibility of the allele calling; classifying genomes from closely related species to identify similar loci; and avoiding adding spurious alleles to a schema when there’s uncertainty about the quality level of the genome assemblies being analyzed.

### Comprehensive allele calling for more accurate and detailed results

**Fig. 3:**
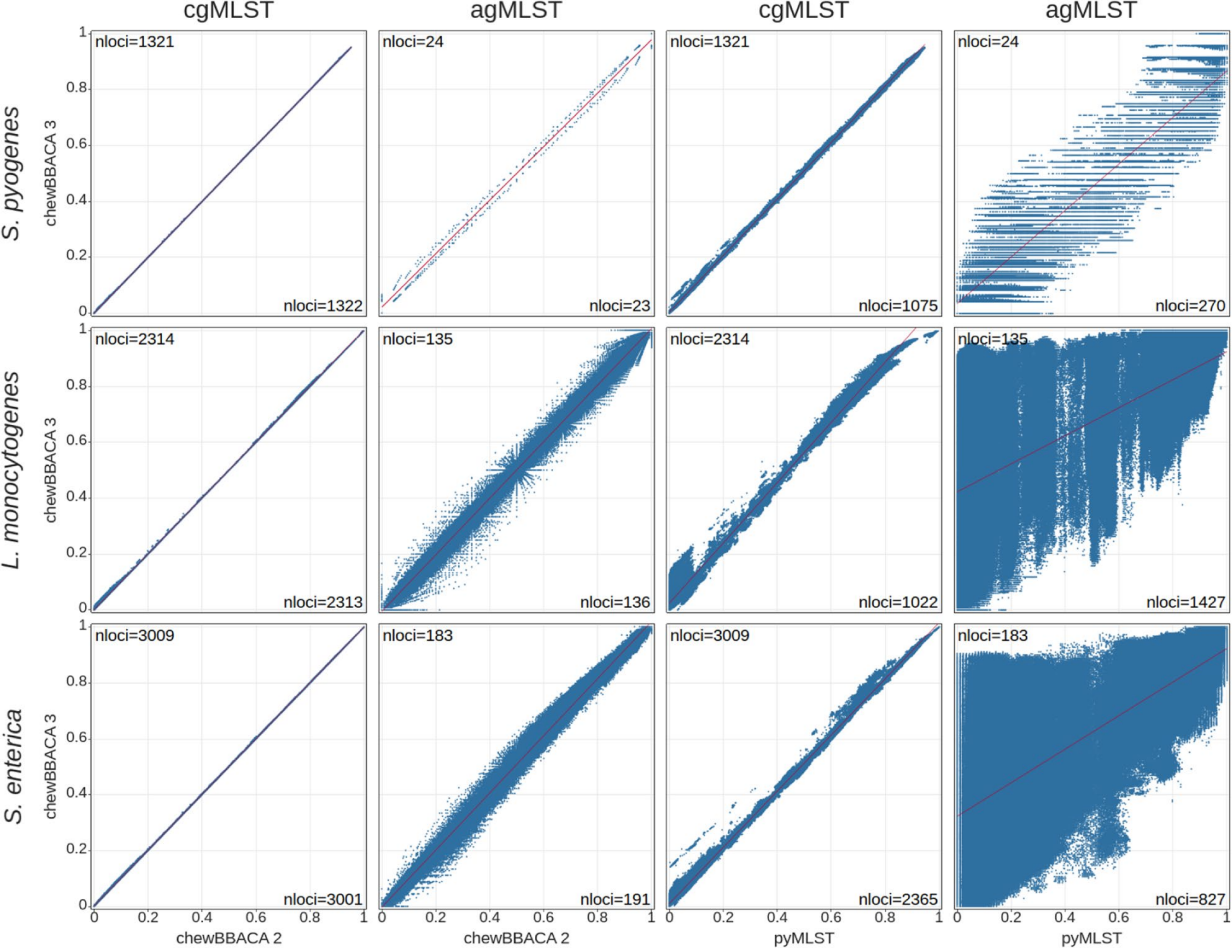
Comparison of the core (cgMLST) and accessory (agMLST) pairwise Jaccard distances. The pairwise Jaccard distances computed based on chewBBACA 3’s allele calling results for the complete datasets (*n* = 16,384 genomes) of *Streptococcus pyogenes*, *Listeria monocytogenes*, and *Salmonella enterica* were compared against the pairwise distances computed from chewBBACA 2’s and pyMLST’s results. The regression lines are displayed in red. The number of core or accessory loci determined based on chewBBACA 3’s results are shown in the top-left corner of the plot area. The number of core or accessory loci determined based on chewBBACA 2’s or pyMLST’s results are shown in the bottom-right corner of the plot area

We evaluated the allele calling results for the complete dataset of each of the three species chosen (consisting of 16,384 genomes) to measure the comprehensiveness of chewBBACA 3’s results and compare it against chewBBACA 2 and pyMLST. Results were compared at the core and accessory genome levels, based on a locus presence threshold of 95%, of the cgMLST schemas defined above. Concordance was measured by comparing the pairwise Jaccard distances computed based on the allelic profiles. The core and accessory loci sets determined based on chewBBACA 3’s and chewBBACA 2’s results were highly similar, sharing over 99% and 95% of the loci at the core and accessory levels, respectively (Additional file 2: Table S11). The pairwise Jaccard distances were strongly correlated and near the identity line, indicating high concordance between the results (Fig. 3), with the pairwise allelic distances computed by both tools differing by 0 to 6 differences on average (Additional file 1: Fig. S14). The core loci sets determined based on pyMLST’s results were considerably smaller, containing 42% to 80% of the schema loci, compared to over 94% for chewBBACA 3. The reduced number of core loci identified by pyMLST is related to an inconsistent identification of some loci in each species. This is partly due to the default identity and coverage thresholds used by pyMLST, which are more stringent than the default BSR threshold used by chewBBACA and do not allow for the same degree of allele sequence variability. Moreover, pyMLST uses a single representative allele per locus to search for matches, whereas chewBBACA 3 can add new representative alleles to schemas to better capture locus diversity. pyMLST’s accessory loci sets were 4- to 11-fold larger than chewBBACA 3’s (Additional file 2: Table S11). The accessory pairwise Jaccard distances were weakly correlated, except for *S. pyogenes*, and the pairwise allelic distances differed by 49 to 141 differences on average. While chewBBACA 2 generates highly comparable results to chewBBACA 3, pyMLST yields considerably different loci sets and pairwise distances, indicating it is not easily comparable to chewBBACA 3. This highlights the importance of the choice of method for cg/wgMLST and how the differences detected and distance thresholds defined by different methods may not be equivalent. chewBBACA 3 classified a similar number of CDSs than chewBBACA 2 for *S. pyogenes* and 1.8% and 0.7% more CDSs for *L. monocytogenes* and *S. enterica*, corresponding to an average of 56 and 33 more CDSs per strain (Fig. 2B and Additional file 2: Table S12). chewBBACA 3 classified 10% to 35% more CDSs than pyMLST, 177 to 1078 more CDSs per strain, on average. chewBBACA 3 and chewBBACA 2 identified over 99% of the schema loci in all strains, while pyMLST identified between 58% and 87% (Fig. 2B). Running chewBBACA 3 in mode 3 provided nearly identical results to the default mode. Modes 1 and 2 classified 4% to 6% fewer CDSs and identified 6% to 7% fewer loci than the default mode, respectively, performing worse if many of the strains’ alleles were not equal or highly similar to the alleles in the schemas (Additional file 1: Fig. S15 and S16).

Compared to chewBBACA 2, chewBBACA 3 identifies special classifications more accurately (Additional file 1: Fig. S17-S19 and Additional file 2: Table S13). The identification of paralogous loci was improved by introducing the PAMA classification (Additional file 1: Fig. S3) for CDSs that match multiple loci and subdividing the NIPH classification into NIPHEM and NIPH to differentiate between multiple exact matches or a combination of exact and inexact matches (Additional file 1: Fig. S2). chewBBACA 3 displays greater sensitivity for detecting multiple matches, leading to more NIPH and NIPHEM classifications than chewBBACA 2, which, in some cases, would detect a single exact match and fail to identify additional inexact matches. The PLOT classification, used by chewBBACA 2 to classify CDSs close to contig ends, was subdivided into PLOT5, PLOT3 and LOTSC to indicate if a CDS is close to the 5’-end, 3’-end, or both (Additional file 1: Fig. S1). New output files include the genomic coordinates for the CDSs predicted for all input genomes and relevant classification statistics per genome and locus. The DNA sequences of the CDSs assigned special classifications or not classified can be stored in FASTA files by providing the *--output-missing* and *--output-unclassified* options, respectively. These changes improve the granularity of the results to facilitate downstream analyses, such as identifying low-quality inputs, paralogous loci, more divergent alleles, and potential new loci to add to schemas.

Another known issue when using cg/wgMLST approaches is that allelic profiles generated with schemas that do not share the same allele nomenclature are not directly comparable. To enable the comparison of results generated with different schemas, chewBBACA 3 includes the *--hash-profiles* option that hashes allele sequences to generate hashed allelic profiles. Since the same allele sequence will always result in the same hash value, the allelic profiles can be compared independently of the nomenclatures used by the schemas allowing also greater data privacy.

### Interactive reports for comprehensive wg/cgMLST schema and allele call results analyses

The schemas and allele calling results generated by chewBBACA 3 can be a source of valuable data for in-depth analyses that explore the loci diversity captured by a schema and the relatedness of strains of interest. We developed modules that enable a local, scalable and comprehensive analysis of cg/wgMLST schemas and results through interactive reports to support users in performing common downstream analyses to more easily reach an informed decision. To showcase the utility of the reports’ functionalities, we analysed 264 *S. pyogenes emm1* strains, including strains from the recently emerged M1_UK_ and M1_DK_ lineages [43, 44], and describe how some of the reports’ components can help identify relevant features to distinguish the lineages.

**Fig. 4:**
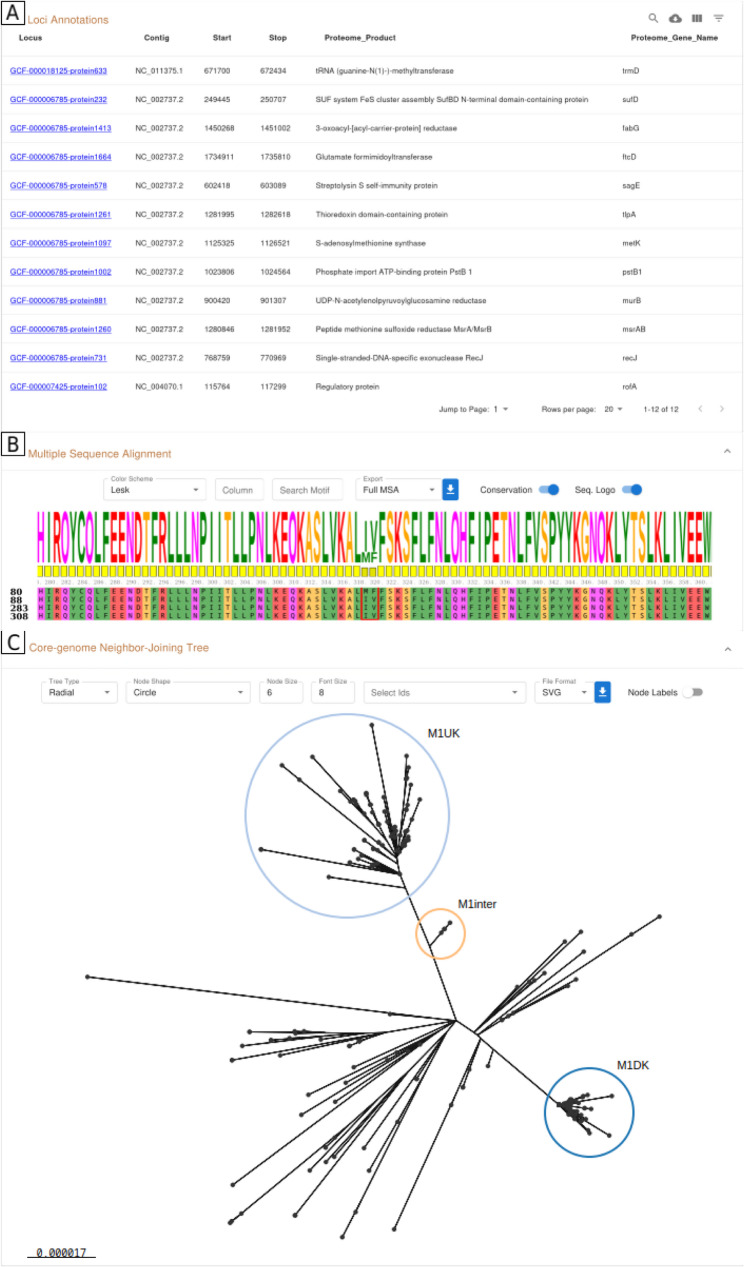
Report components generated for the analysis of the *S. pyogenes* schema and lineage strains. (**A**) Datatable component of the report generated by the *SchemaEvaluator* module including the annotations determined by the *UniprotFinder* module for 12 schema loci containing lineage-defining SNPs for the M1_UK_ lineage. (**B**) Component of the *SchemaEvaluator* module including a Multiple sequence alignment (MSA) of the *rofA* translated alleles identified in the MGAS5005 reference strain (allele 80) and the M1_UK_ strains (alleles 88, 283, and 308). Two amino acid differences caused by two SNPs in the *rofA* alleles of the M1_UK_ strains are highlighted in red. (**C**) Component of the *AlleleCallEvaluator* module including a Neighbor-Joining tree computed with FastTree from the core loci MSA. The groups of strains belonging to the M1_UK_ (light blue), M1_inter_ (light orange), and M1_DK_ (dark blue) lineages are highlighted. The full reports are available on Zenodo [48]

The *SchemaEvaluator* (Additional file 1: Fig. S20) module evaluates cg/wgMLST schemas created with chewBBACA or from external sources to create an interactive report with detailed information about the schema composition. The main page of the report includes charts that allow exploring the number of alleles and the allele size variation per locus. The module accepts a file with loci annotations to facilitate the identification of loci of interest. For example, the annotations determined by the *UniprotFinder* module (Additional file 1: Fig. S21) for the *S. pyogenes* schema can be added to a data table to identify which schema loci have the lineage-defining single-nucleotide polymorphisms (SNPs) of the M1_UK_ (Fig. 4A) and M1_DK_ lineages. Another data table displays the results of the allele integrity analysis, which identifies classes of invalid alleles per locus (e.g. incomplete CDSs, presence of ambiguous bases, absence of start and stop codons, in-frame stop codons, and minimum and locus-specific size thresholds). This can be used to identify problematic loci or loci with unusual size variability. The *--loci-reports* option provides a more detailed analysis of each locus through dedicated locus pages. Each locus page contains charts for the allele size distribution, sequence size per allele and number of DNA alleles for each distinct protein. A multiple sequence alignment (MSA) computed with MAFFT [46] for the translated alleles allows identifying shared regions and differences caused by point mutations or indels. For example, the non-synonymous effect of two SNPs in the *rofA* gene used to define the M1_UK_ lineage can be identified using the MSA by comparing the reference allele with those identified in M1_UK_ strains (Fig. 4B). The guide tree created by MAFFT is displayed with Phylocanvas.gl [47] to help identify groups of similar or divergent alleles. To provide a convenient way to identify and copy the DNA and protein sequences of the alleles, users can use the *--add-sequences* option, which makes the DNA and protein sequences for each locus available through the locus pages.

A report with a detailed analysis of the allele calling results is obtained by running the *AlleleCallEvaluator* module (Additional file 1: Fig. S22). The report includes data tables with summary statistics and bar charts with the classification counts per strain and locus to explore the classification results and aid in identifying low-quality genomes (e.g. misassembled or contaminated genomes) and problematic loci (e.g. loci with a high number of special classifications). An interactive analysis of loci presence-absence is performed through a heatmap component that enables identifying the set of core loci or loci specific to certain groups of strains. Similar strains can be identified through another heatmap component that displays the matrix of pairwise core allelic distances and enables searching for similar strains based on a distance threshold. The last component in the report displays a Neighbor-Joining tree computed with FastTree 2 [49] from the core loci MSA. This component allows exploring phylogenetic relationships to identify groups of similar strains. For instance, the NJ tree from the analysis of the *S. pyogenes* strains allows identifying the groups of strains corresponding to each lineage of the M1 group (Fig. 4C).

The reports’ components include features to sort, select, search, and export data in tabular format, in the case of data tables, or as SVG files, in the case of charts or trees. Some files necessary to create the components, such as the ones containing the matrix of pairwise core allelic distances and the core loci MSA, are provided in the report’s folder to allow users to perform custom analyses if desired. The reports are easily shared by simply compressing the report’s folder and sharing the resulting archive.

### chewBBACA 3 provides increased resolution for the discrimination of closely related strains

To evaluate the performance of chewBBACA 3 for the discrimination of closely related strains, we performed allele calling and computed the inter- and intra-lineage pairwise allelic distances for the 264 *S. pyogenes emm1* strains grouped by lineage analysed in the previous section and 501 *S. pneumoniae* serotype 3 strains grouped based on multiple classification systems from a previous study [50]. To maximize the identification of lineage-specific loci, the schemas used in these analyses were created by the CreateSchema module of each chewBBACA version with each species’ entire dataset. The databases for pyMLST were created with the representative alleles of the loci identified by chewBBACA 3.

For the *S. pyogenes* strains, chewBBACA 3 identified, on average, between 65 and 67 more loci than chewBBACA 2 at the intra-lineage level and between 64 and 66 more loci at the inter-lineage level (Additional file 1: Fig. S23). pyMLST identified a similar number of loci as chewBBACA 3 (Additional file 1: Fig. S23), which is not unexpected since its database includes the same loci and representative alleles used by chewBBACA 3. This result also shows that using a set of representative alleles more closely related to the strains’ dataset does improve loci identification with pyMLST as suggested above. For the *S. pneumoniae* strains, chewBBACA 3 identified more loci than chewBBACA 2 and pyMLST at both the intra- and inter-cluster level (on average, 66 to 85 more loci than chewBBACA 2, and 93 to 147 more than pyMLST) (Additional file 1: Fig. S24-S27). The number of additional loci classified by chewBBACA 3 corresponds to an increased resolution of up to 3.5% and 5.7% for the *S. pyogenes* and *S. pneumoniae* datasets, respectively.

The differences relative to chewBBACA 2 are mostly due to the larger size of the schemas created by chewBBACA 3 (an additional 128 loci for *S. pyogenes* and 185 loci for *S. pneumoniae*), which, as we have previously shown, identifies more distinct loci than chewBBACA 2 during schema creation. If we include the loci assigned special classifications by chewBBACA 3 as potential additional loci, chewBBACA 3 identifies, on average, 23 to 29 additional loci in each strain of the *S. pyogenes* dataset and 143 to 216 additional loci in the *S. pneumoniae* dataset compared to pyMLST (Additional file 1: Fig. S23-S27). This highlights that the number of cases where loci presence cannot be confidently inferred using chewBBACA 3’s criteria may represent a considerable number of potential differences, and should, especially when analysing closely related strains, be carefully evaluated to more accurately differentiate strains and identify features of potential interest.

We also determined if, in addition to identifying more loci, the pairwise distances computed based on chewBBACA 3 are different from the ones computed based on chewBBACA 2 and pyMLST. The number of differences of the pairwise allelic comparisons computed from the results of all tools were similar at the intra-cluster level (Additional file 1: Fig. S28-S32), with chewBBACA 3 predicting, an average of -3 to 6 and -0.5 to 40 additional differences than chewBBACA 2 and pyMLST, respectively. The inter-cluster differences vary more, with chewBBACA 3 identifying, an average of between 0.5 and 40 and 3 to 150 additional differences compared to chewBBACA 2 and pyMLST, respectively (Additional file 1: Fig. S28-S32). These smaller distance differences relative to the gains in number of loci indicate that the additional loci identified by chewBBACA 3 have alleles that are shared by most strains. Distance differences below 0 correspond mostly to strains for which chewBBACA 3 assigns a higher number of special classifications, which are identified by the other tools as valid alleles. Due to the uncertainty in determining loci presence or allele identity, the special classifications assigned by chewBBACA 3 are excluded during the computation of the pairwise distances, potentially resulting in an overall smaller number of differences if other tools classify these loci as having valid alleles.

## Conclusions

chewBBACA 3 constitutes an efficient, scalable, and comprehensive solution for wg/cgMLST. The options it provides for schema setup enable users to quickly create schemas from larger collections of genome assemblies or CDS data to capture more of the diversity of a bacterial species, or to adapt or import existing schemas created in other platforms or available in Chewie-NS to promote interoperability. The combination of alignment-based and alignment-free approaches allows for efficient and accurate allele calling, making it suitable for integration into workflows that process sample batches of any size, from sequential processing of single samples to vast genome collections for species-level population analyses. chewBBACA 3 classifies more schema loci and CDSs than the compared methods, potentially providing superior strain discrimination for surveillance and outbreak investigation. The high level of agreement with chewBBACA 2’s results, while providing expanded classifications and richer results, facilitates the transition to the latest chewBBACA version. Comparisons with other wg/cgMLST methods should take into account that algorithmic differences between methods, parameter values, and input data quality can greatly affect the resolution and accuracy of the results, which might hinder results comparison and in some cases even lead to fundamentally different conclusions. The reports for schema and allele call evaluation allow a comprehensive and local analysis of locus diversity and strain similarity, enabling scalable and private analyses of the results and reducing the need to combine several tools or develop custom solutions to more fully explore the potential of wg/cgMLST schemas. The integration of chewBBACA 3 into wg/cgMLST workflows will help to further democratize wg/cgMLST by providing broader access to large-scale and detailed analyses to perform focused population studies or facilitate reaching an informed decision in outbreak or transmission investigations.

## Availability and requirements

Project name: chewBBACA 3.

Project home page: https://github.com/B-UMMI/chewBBACA.

Project documentation: https://chewbbaca.readthedocs.io/en/latest/index.html.

Operating system(s): Linux and macOS.

Programming language: Python > = 3.8.

Other requirements: BLAST+ >= 2.9.0, pyrodigal > = 3.0.0, numpy ~ = 1.24.3, scipy ~ = 1.10.1, biopython > = 1.79, plotly > = 5.8.0, SPARQLWrapper > = 2.0.0, requests > = 2.27.1, pandas > = 1.5.1.

License: GPL-3.0.

Any restrictions to use by non-academics: None.

## Supplementary Information

Additional file 1. This additional file includes the supplementary figures (Fig. S1 to S32). - Fig. S1: PLOT3, PLOT5 and LOTSC classifications. Fig. S2: NIPH and NIPHEM classifications. Fig. S3: PAMA classification. Fig. S4: ASM and ALM classifications. Fig. S5: Diagram of the CreateSchema module. Fig. S6: Diagram of the AlleleCall module. Fig. S7: Sequence hashing and modified polyline encoding. Fig. S8: Diagram of the PrepExternalSchema module. Fig. S9: Diagram of the DownloadSchema module. Fig. S10: Diagram of the LoadSchema module. Fig. S11: Diagram of the SyncSchema module. Fig. S12: Diagram of the ExtractCgMLST module. Fig. S13: Runtime and peak memory usage for the four execution modes available in chewBBACA 3. Fig. S14: Pairwise allelic distances differences. Fig. S15: Proportion of CDSs classified per execution mode for each species’ datasets. Fig. S16: Proportion of schema loci classified per execution mode for each species’ datasets. Fig. S17: Classifications counts for the complete dataset of S. pyogenes per tool. Fig. S18: Classifications counts for the complete dataset of L. monocytogenes per tool. Fig. S19: Classifications counts for the complete dataset of S. enterica per tool. Fig. S20: Diagram of the SchemaEvaluator module. Fig. S21: Diagram of the UniprotFinder module. Fig. S22: Diagram of the AlleleCallEvaluator module. Fig. S23: Number of additional loci found by chewBBACA 3 for 264 S. pyogenes strains. Fig. S24: Number of additional loci found by chewBBACA 3 for 501 S. pneumoniae strains grouped by Sequence Type. Fig. S25: Number of additional loci found by chewBBACA 3 for 501 S. pneumoniae strains grouped by Global Pneumococcal Sequence Cluster. Fig. S26: Number of additional loci found by chewBBACA 3 for 501 S. pneumoniae strains grouped by Azarian et al. clade. Fig. S27: Number of additional loci found by chewBBACA 3 for 501 S. pneumoniae strains grouped by Kwun et al. clade. Fig. S28: Intra- and inter-cluster distance differences for 264 S. pyogenes strains. Fig. S29: Intra- and inter-cluster distance differences for 501 S. pneumoniae strains grouped by sequence type. Fig. S30: Intra- and inter-cluster distance differences for 501 S. pneumoniae strains grouped by Global Pneumococcal Sequence Cluster. Fig. S31: Intra- and inter-cluster distance differences for 501 S. pneumoniae strains grouped by Azarian et al. clade. Fig. S32: Intra- and inter-cluster distance differences for 501 S. pneumoniae strains grouped by Kwun et al. clade.

Additional file 2. This additional file includes the supplementary tables (Tables S1 to S13). Table S1. Runtime (in minutes, min) and peak memory usage (in megabytes, MB) values for the creation of the schema seeds with chewBBACA 2 and chewBBACA 3 based on the complete genomes for each species. Table S2. Number of loci in the schema seeds, number of loci shared between schema seeds based on the BSR, minimizers, and both, and percentage of loci in schema seeds created by chewBBACA 2 that are shared with the schema seeds created by chewBBACA 3. Table S3. Runtime (in minutes, min) and peak memory usage (in megabytes, MB) for the adaptation of the schemas downloaded from cgMLST.org with chewBBACA 2 and chewBBACA 3. Table S4. Number of loci in the schemas download from cgMLST.org, number of loci in the adapted schemas, and number of loci whose diversity is not completly captured by the selected representative alleles. Table S5. Number of loci in the wgMLST and cgMLST schemas, and number of alleles in the cgMLST schemas after performing allele calling with each tool with the complete genomes. Table S6. Mean runtime values in minutes for the allele calling of each species’ datasets with chewBBACA 3, chewBBACA 2 and pyMLST. Table S7. Mean values for the total number of coding sequences (CDSs), distinct number of CDSs, and percentage of total CDSs that are distinct for each species’ datasets. Table S6. Mean peak memory usage values in megabytes for the allele calling of each species’ datasets with chewBBACA 3, chewBBACA 2 and pyMLST. Table S9. Mean runtime values in minutes for the allele calling of each species’ datasets with chewBBACA 3’s four execution modes. Table S10. Mean peak memory usage values in megabytes for the allele calling of each species’ datasets with chewBBACA 3’s four execution modes. Table S11. Number of loci in each species’ cgMLST schemas, number of core and accessory loci determined based on each tool’s results and number of core and accessory loci determined based on chewBBACA 2’s and pyMLST’s results that are shared with the sets of core and accessory loci determined based on chewBBACA 3’s results. Table S12. Total number of coding sequences (CDSs) predicted by Pyrodigal for each species’ complete dataset, total number of CDSs classified by each tool, percentage of the total CDSs classified by each tool and average number of CDSs classified per strain. Table S13. Special classification counts for each species’ complete dataset per tool.

## Data Availability

The datasets, schemas and databases created and used with chewBBACA 3, chewBBACA 2, and pyMLST, and all results generated for each section are available on Zenodo: 10.5281/zenodo.14637858. The supplementary figures and tables are included in the additional files.

## Abbreviations

ANI: Average nucleotide identity
BLASTp: Protein BLAST
BSR: BLAST Score Ratio
CDS: Coding DNA sequence
ECDC: European Centre for Disease Prevention and Control
EFSA: European Food Safety Authority
ENA: European Nucleotide Archive
GbG: Gene-by-gene
MSA: Multiple sequence alignment
NCBI: National Center for Biotechnology Information
NJ: Neighbor-Joining
SNP: Single nucleotide polymorphism
SNV: Single nucleotide variant
ST: Sequence Type
wg/cgMLST: Whole genome and core genome multilocus sequence typing
WGS: Whole genome sequencing
