# Supplementary material for: chewBBACA 3: lowering the barrier for scalable and detailed whole- and core-genome multilocus sequence typing
Source: Genome Med. 2026 Mar 24;18:51. doi: 10.1186/s13073-026-01625-x (PMC13134279; doi:10.1186/s13073-026-01625-x)
Supplement: Supplementary file 1 — Additional file 1. This additional file includes the supplementary figures (Fig. S1 to S32). - Fig. S1: PLOT3, PLOT5 and LOTSC classifications. Fig. S2: NIPH and NIPHEM classifications. Fig. S3: PAMA classification. Fig. S4: ASM and ALM classifications. Fig. S5: Diagram of the CreateSchema module. Fig. S6: Diagram of the AlleleCall module. Fig. S7: Sequence hashing and modified polyline encoding. Fig. S8: Diagram of the PrepExternalSchema module. Fig. S9: Diagram of the DownloadSchema module. Fig. S10: Diagram of the LoadSchema module. Fig. S11: Diagram of the SyncSchema module. Fig. S12: Diagram of the ExtractCgMLST module. Fig. S13: Runtime and peak memory usage for the four execution modes available in chewBBACA 3. Fig. S14: Pairwise allelic distances differences. Fig. S15: Proportion of CDSs classified per execution mode for each species’ datasets. Fig. S16: Proportion of schema loci classified per execution mode for each species’ datasets. Fig. S17: Classifications counts for the complete dataset of S. pyogenes per tool. Fig. S18: Classifications counts for the complete dataset of L. monocytogenes per tool. Fig. S19: Classifications counts for the complete dataset of S. enterica per tool. Fig. S20: Diagram of the SchemaEvaluator module. Fig. S21: Diagram of the UniprotFinder module. Fig. S22: Diagram of the AlleleCallEvaluator module. Fig. S23: Number of additional loci found by chewBBACA 3 for 264 S. pyogenes strains. Fig. S24: Number of additional loci found by chewBBACA 3 for 501 S. pneumoniae strains grouped by Sequence Type. Fig. S25: Number of additional loci found by chewBBACA 3 for 501 S. pneumoniae strains grouped by Global Pneumococcal Sequence Cluster. Fig. S26: Number of additional loci found by chewBBACA 3 for 501 S. pneumoniae strains grouped by Azarian et al. clade. Fig. S27: Number of additional loci found by chewBBACA 3 for 501 S. pneumoniae strains grouped by Kwun et al. clade. Fig. S28: Intra- and inter-cluster distance differences for 264 S. [file 13073_2026_1625_MOESM1_ESM.pdf]

Additional file 1. Supplementary figures

This file includes the supplementary figures cited in the main manuscript.
It includes the following figures:

- **Fig. S1**: PLOT3, PLOT5 and LOTSC classifications.
- **Fig. S2**: NIPH and NIPHEM classifications.
- **Fig. S3**: PAMA classification.
- **Fig. S4**: ASM and ALM classifications.
- **Fig. S5**: Diagram of the *CreateSchema* module.
- **Fig. S6**: Diagram of the *AlleleCall* module.
- **Fig. S7**: Sequence hashing and modified polyline encoding.
- **Fig. S8**: Diagram of the *PrepExternalSchema* module.
- **Fig. S9**: Diagram of the *DownloadSchema* module.
- **Fig. S10**: Diagram of the *LoadSchema* module.
- **Fig. S11**: Diagram of the *SyncSchema* module.
- **Fig. S12**: Diagram of the *ExtractCgMLST* module.
- **Fig. S13**: Runtime and peak memory usage for the four execution modes available in chewBBACA 3.
- **Fig. S14**: Pairwise allelic distances differences.
- **Fig. S15**: Proportion of CDSs classified per execution mode for each species' datasets.
- **Fig. S16**: Proportion of schema loci classified per execution mode for each species' datasets.
- **Fig. S17**: Classifications counts for the complete dataset of *S. pyogenes* per tool.
- **Fig. S18**: Classifications counts for the complete dataset of *L. monocytogenes* per tool.
- **Fig. S19**: Classifications counts for the complete dataset of *S. enterica* per tool.
- **Fig. S20**: Diagram of the *SchemaEvaluator* module.
- **Fig. S21**: Diagram of the *UniprotFinder* module.
- **Fig. S22**: Diagram of the *AlleleCallEvaluator* module.
- **Fig. S23**: Number of additional loci found by chewBBACA 3 for 264 *S. pyogenes* strains.
- **Fig. S24**: Number of additional loci found by chewBBACA 3 for 501 *S. pneumoniae* strains grouped by Sequence Type.
- **Fig. S25**: Number of additional loci found by chewBBACA 3 for 501 *S. pneumoniae* strains grouped by Global Pneumococcal Sequence Cluster.
- **Fig. S26**: Number of additional loci found by chewBBACA 3 for 501 *S. pneumoniae* strains grouped by Azarian et al. clade.
- **Fig. S27**: Number of additional loci found by chewBBACA 3 for 501 *S. pneumoniae* strains grouped by Kwun et al. clade.
- **Fig. S28**: Intra- and inter-cluster distance differences for 264 *S. pyogenes* strains.
- **Fig. S29**: Intra- and inter-cluster distance differences for 501 *S. pneumoniae* strains grouped by sequence type.
- **Fig. S30**: Intra- and inter-cluster distance differences for 501 *S. pneumoniae* strains grouped by Global Pneumococcal Sequence Cluster.
- **Fig. S31**: Intra- and inter-cluster distance differences for 501 *S. pneumoniae* strains grouped by Azarian et al. clade.
- **Fig. S32**: Intra- and inter-cluster distance differences for 501 *S. pneumoniae* strains grouped by Kwun et al. clade.

Fig. S1. PLOT5, PLOT3 and LOTSC classifications.

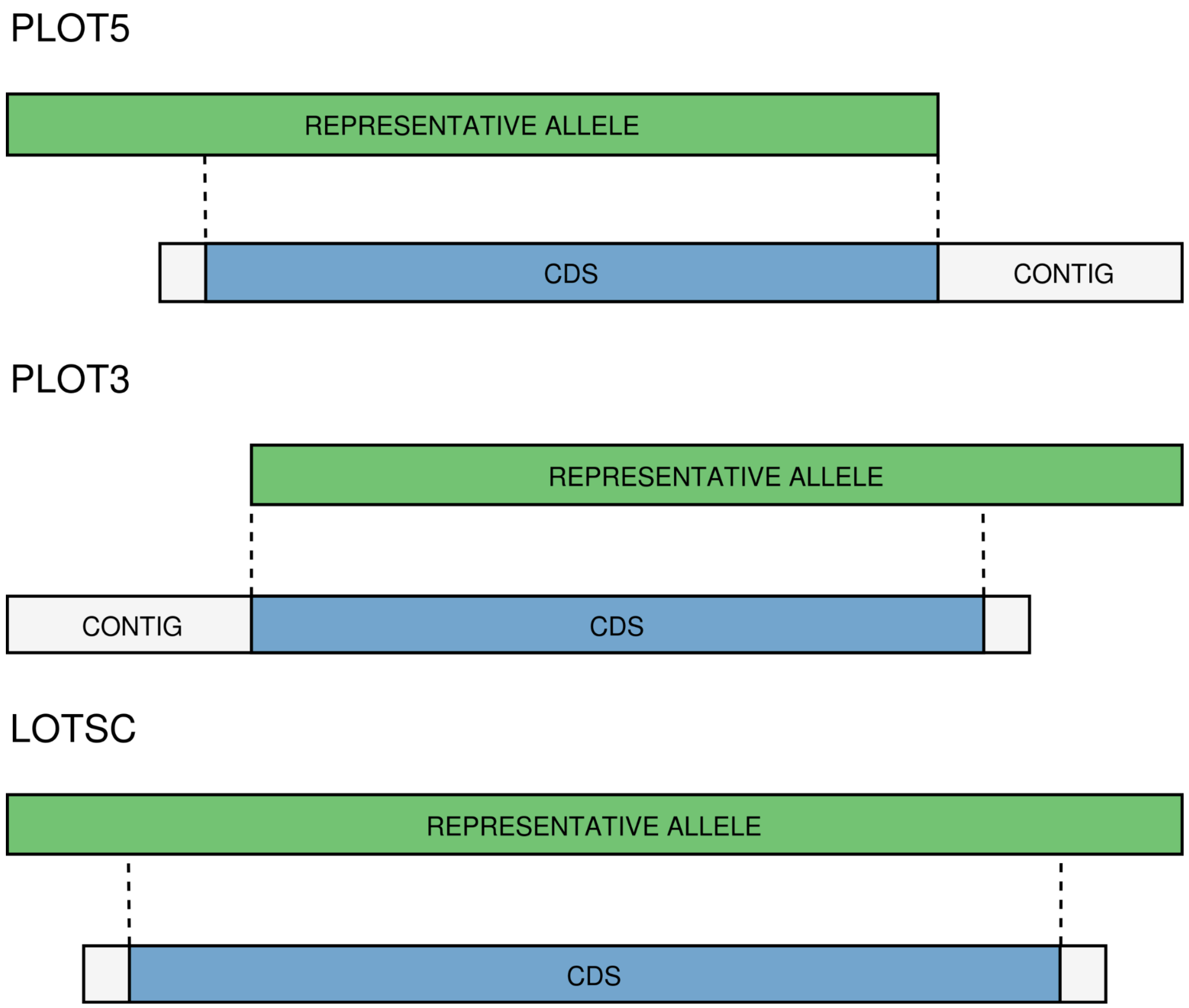

Fig. S1. The PLOT3, PLOT5 and LOTSC classifications are related to the position of CDSs in the genomic contigs. PLOT5 and PLOT3 (Possible Locus On the Tip) - a CDS is classified as PLOT5 or PLOT3 if it is close to the contig 5'- or 3'-end and if the unaligned portion of the matched representative allele exceeds the contig end. LOTSC - a CDS is classified as LOTSC if the matched representative allele is bigger than the contig containing the CDS.

Fig. S2. NIPH and NIPHEM classifications.

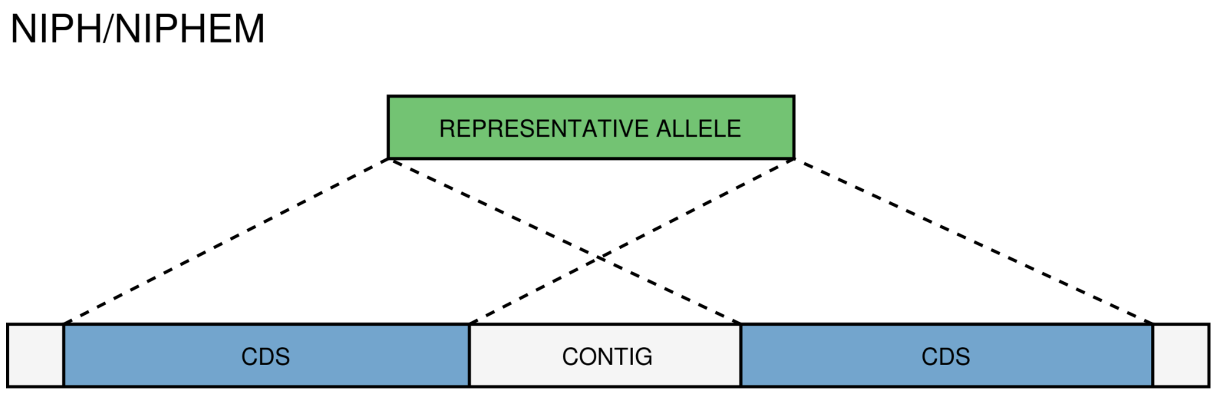

Fig. S2. The NIPH and NIPHEM classifications are assigned when multiple CDSs from the same genome match the same schema locus. NIPH (Non-Informative Paralogous Hit) - assigned when multiple CDSs from the same genome match a single locus. NIPHEM (Non-Informative Paralogous Hit Exact Match) - assigned when multiple CDSs from the same genome are exact matches to alleles of a single locus.

Fig. S3. PAMA classification.

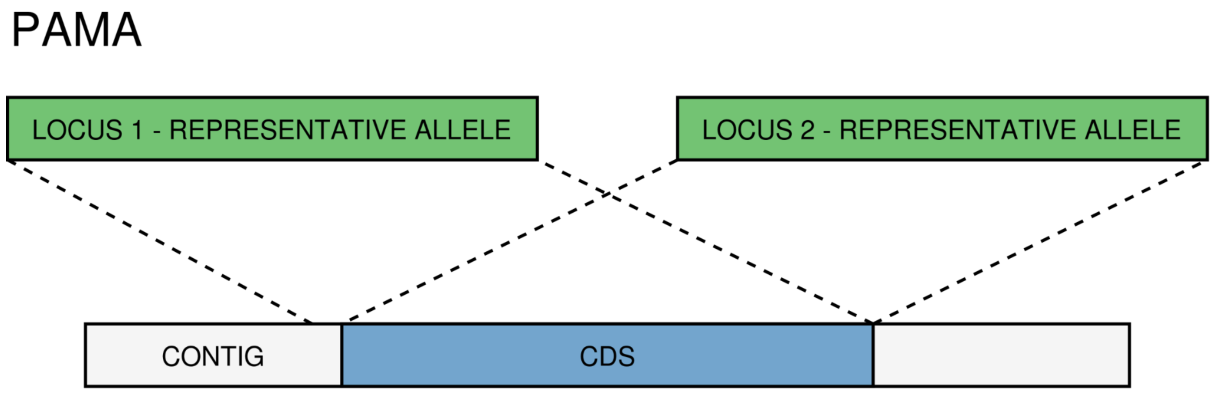

Fig. S3. The PAMA (PAralogous MAtch) classification is assigned when a single CDS from a genome matches multiple schema loci.

Fig. S4. ASM and ALM classifications.

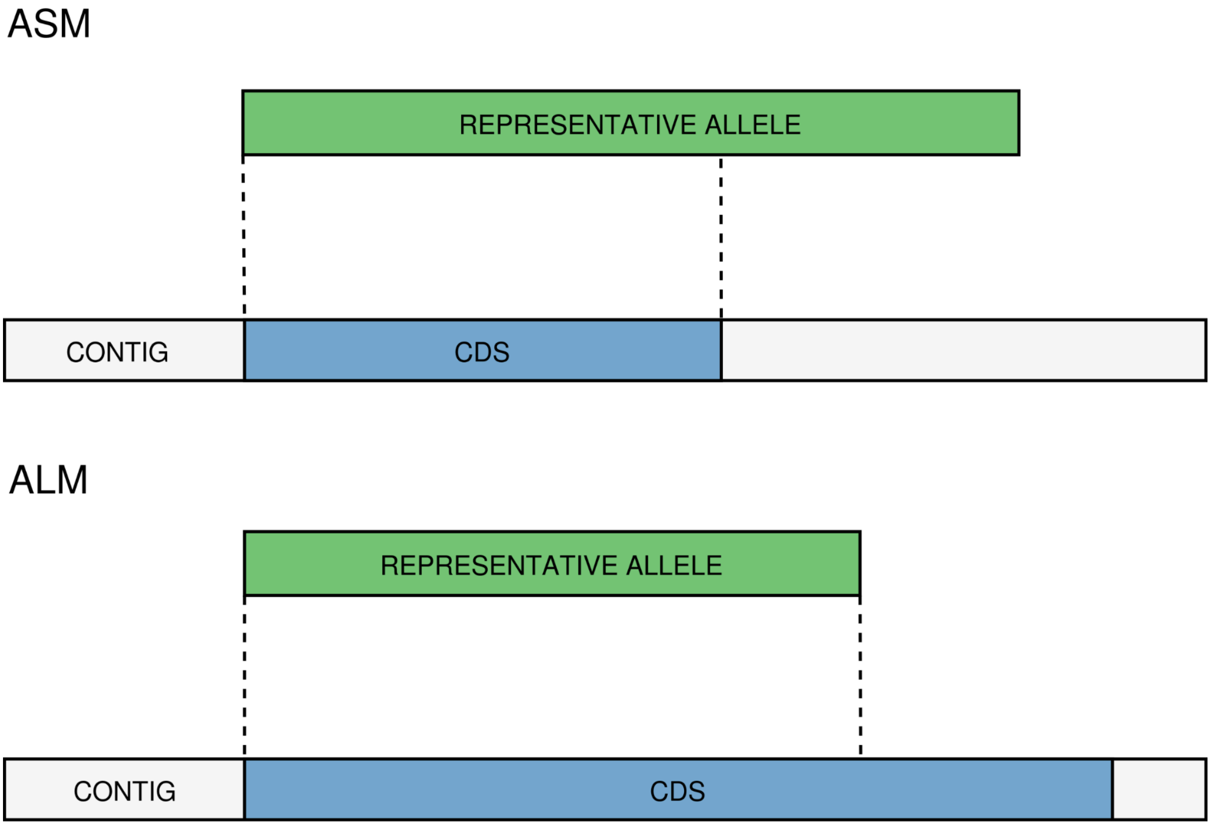

Fig. S4. The ASM (Allele Smaller than Mode) and ALM (Allele Larger than Mode) classifications are assigned when the size of a CDS that matches a schema locus is below or above the locus size variation interval, respectively. The default behaviour is to assign these classifications to alleles that are 20% shorter or longer than the locus allele size mode.

Fig. S5. Diagram of the *CreateSchema* module.

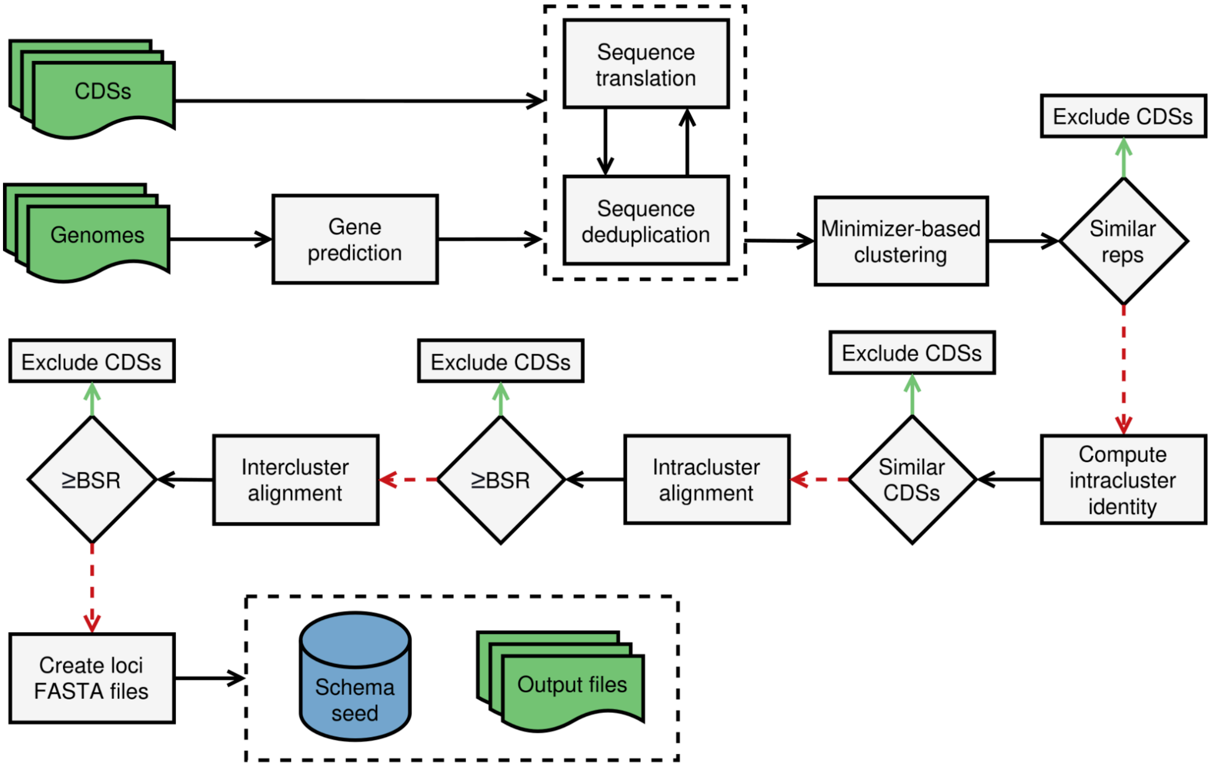

Fig. S5. The *CreateSchema* module creates a schema seed based on a set of FASTA files with genome assemblies or CDSs. If genome assemblies are given, the process starts by predicting CDSs for each genome using Pyrodigal. The CDSs identified in the input files are deduplicated and translated, followed by a second deduplication step to determine the set of distinct translated CDSs. The distinct translated CDSs are clustered based on the proportion of minimizers shared with representative CDSs. The largest or one of the largest CDSs is selected as the first representative CDS. New representative CDSs are selected when CDSs share a low proportion (<0.2) of minimizers with any of the chosen representative CDSs. Non-representative CDSs that share a proportion of minimizers ≥ 0.9 with the cluster representative are considered to correspond to the same locus and are excluded from the analysis. The proportion of shared minimizers between non-representative CDSs is determined to exclude CDSs sharing a proportion of minimizers ≥ 0.9 with larger CDSs. Intracluster and intercluster alignment with BLASTp enable identifying and excluding CDSs similar to representative or larger non-representative CDSs based on a BLAST Score Ratio (BSR) ≥ 0.6. Each remaining CDS is considered to be an allele of a distinct locus. The process ends by creating a schema seed, which includes one FASTA file containing a single representative allele per distinct locus identified in the analysis. Green document icons represent input FASTA files and output files. Grey rectangle icons represent analysis steps. Diamond icons represent conditional statements, with green arrows used when the condition is met and red dashed arrows otherwise. The blue cylinder icon represents the schema seed created by the *CreateSchema* module.

Fig. S6. Diagram of the AlleleCall module.

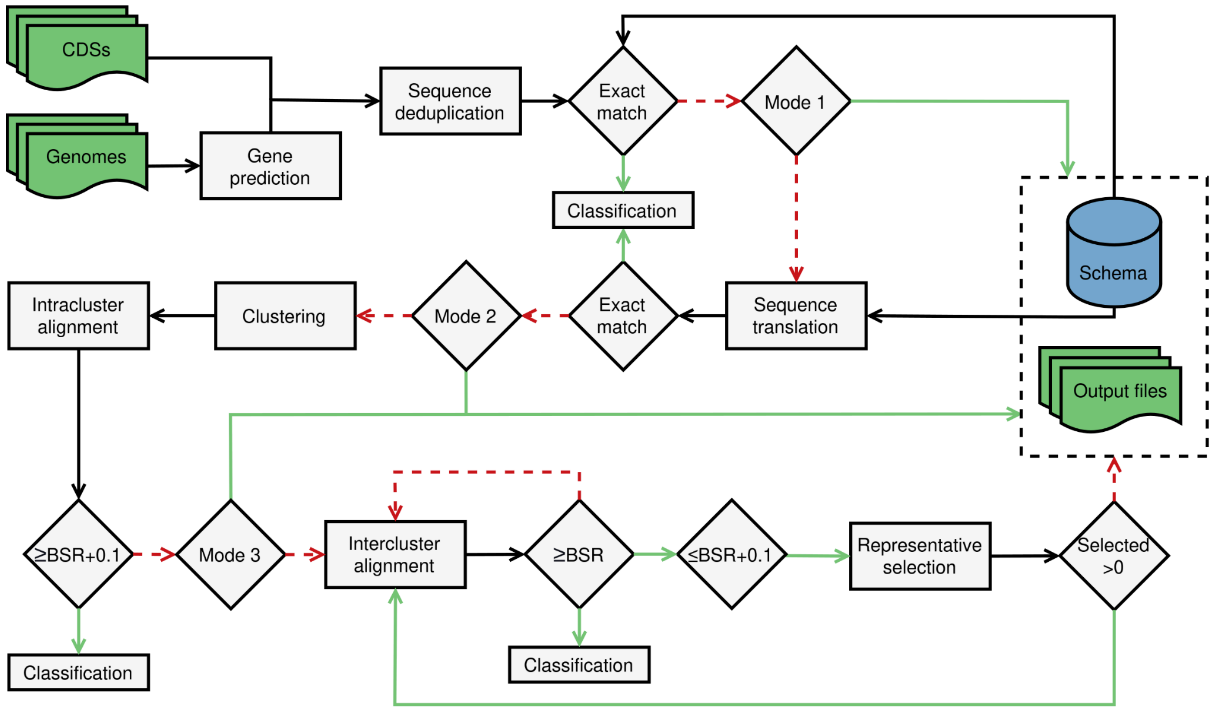

Fig. S6. The *AlleleCall* module determines the allelic profiles for strains of interest. The process accepts FASTA files with genome assemblies or CDSs. If genome assemblies are given, the process starts by predicting CDSs for each genome using Pyrodigal. The CDSs identified in the input files are deduplicated and compared against the schema alleles to find and classify exact matches at the DNA level. If the process runs in mode 1, the results are evaluated to write the output files and exit. Otherwise, the CDSs that do not match any schema alleles at the DNA level are translated and matched against the translated schema alleles to find exact matches at the protein level. If the process runs in mode 2, the results are evaluated to write the output files, add new alleles to the schema and exit. Otherwise, the CDSs not classified through exact matching are compared against the schema representative alleles through minimizer-based clustering to identify CDSs that share a proportion of minimizers ≥ 0.2 with the representative alleles. Each cluster's representative allele is aligned against the clustered CDSs with BLASTp to classify CDSs based on the defined BLAST Score Ratio (BSR) value plus 0.1. At this point, if the process runs in mode 3, the results are evaluated to write the output files, add new alleles to the schema and exit. Otherwise, the representative alleles are aligned against the remaining unclassified CDSs to classify them based on the defined BSR value and identify new representative alleles whose BSR is not above the defined BSR value plus 0.1. If the process finds new representative alleles, it aligns them against the unclassified CDSs to find new matches. This process repeats until no new representative alleles are identified. When no new representative alleles are found, the process evaluates the results to create the output files, add new alleles to the schema, and exit. Green document icons represent input FASTA files and output files. Grey rectangle icons represent analysis steps. Diamond icons represent conditional statements, with green arrows used when the condition is met and red dashed arrows otherwise. The blue cylinder icon represents a schema.

Fig. S7. Sequence hashing and modified polyline encoding.

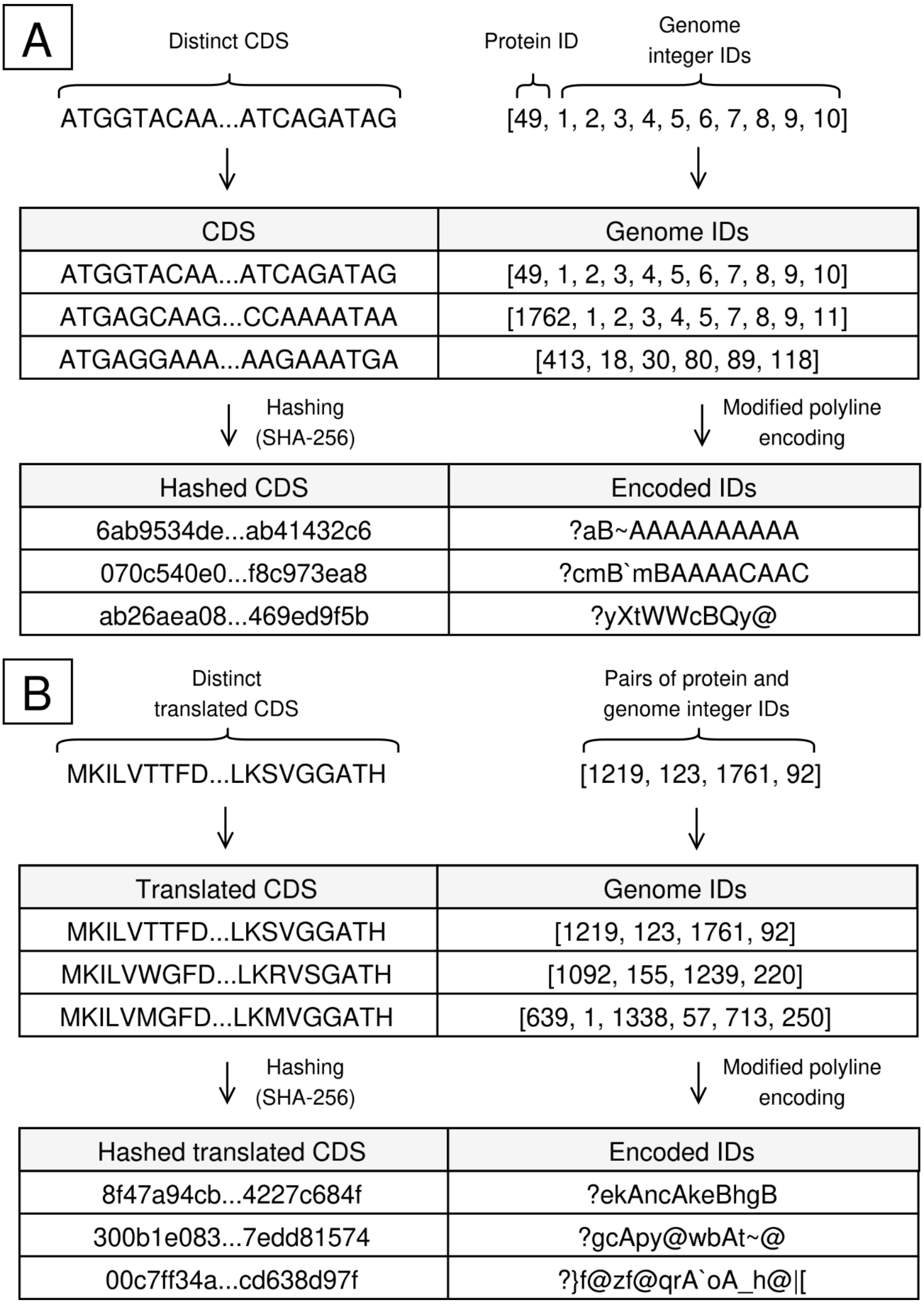

Fig. S7. (A) Each distinct CDS identified in the input genomes is hashed with the SHA-256 algorithm implemented in Python's *hashlib* library. The hash digest is obtained through the *hexdigest* method and mapped to the list of integer identifiers for the genomes containing the CDS encoded with modified polyline encoding. (B) After sequence translation and deduplication, each distinct translated CDS is hashed with the SHA-256 algorithm and the hash digest is mapped against lists with pairs of protein and genome identifiers used to identify each distinct CDS coding for the protein encoded with modified polyline encoding. The modified polyline encoding is applied to reduce the memory used to retain the data in-memory during the process, drastically reducing peak memory usage when processing large datasets. The Python dictionaries created to map the hashes to the lists of identifiers allow quick identification and classification of exact and inexact matches.

Fig. S8. Diagram of the *PrepExternalSchema* module.

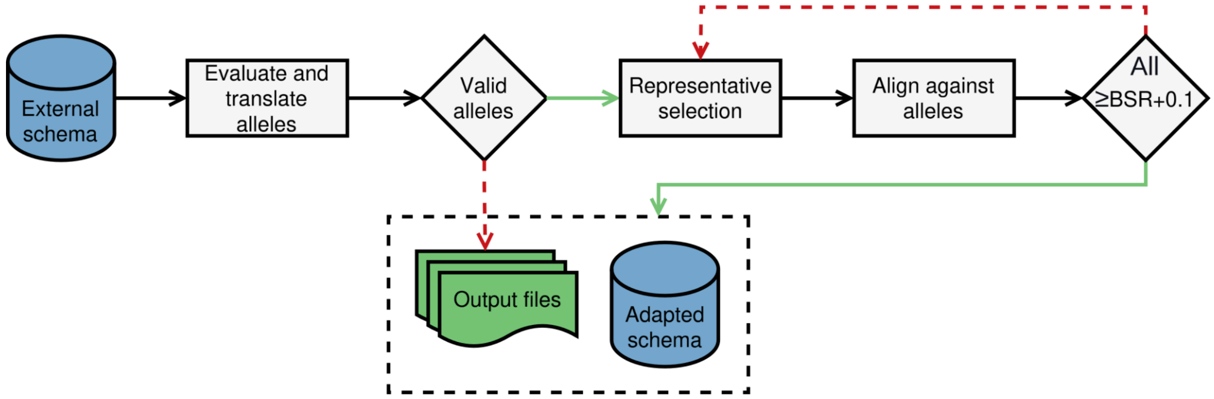

Fig. S8. The *PrepExternalSchema* module adapts schemas created with other wg/cgMLST tools or available on external platforms for usage with chewBBACA 3. The process starts by validating and translating the alleles in the external schema. Incomplete (i.e. size not multiple of 3) and invalid (i.e. missing the start or stop codons, or containing in-frame stop codons) alleles, alleles containing ambiguous bases or smaller than the specified minimum length value, are excluded. For each locus that has valid alleles, the process selects the largest or one of the largest alleles as the first representative allele. The representative is aligned against the locus' alleles with BLASTp to compute the BSR for each alignment. If all the BSR values are above the specified BSR plus 0.1, it is considered that the representative allele can adequately capture the diversity of the locus. Otherwise, new representative alleles are selected from those with a BSR above the specified BSR but below that value plus 0.1 to align against the locus' alleles and determine if the set of representative alleles selected captures the locus diversity adequately. Representative selection is repeated until all locus' alleles have a BSR above the specified value plus 0.1 with at least one of the selected representative alleles. The valid and selected representative alleles are written to FASTA files to create a schema compatible with chewBBACA. The list of invalid alleles, the list of loci excluded from the adapted schema due to having no valid alleles, and the number of total alleles and representative alleles per locus in the adapted schema are stored in output files. The green document icons represent output files. Grey rectangle icons represent analysis steps. Diamond icons represent conditional statements, with green arrows used when the condition is met and red dashed arrows otherwise. The blue cylinder icons represent schemas.

Fig. S9. Diagram of the *DownloadSchema* module.

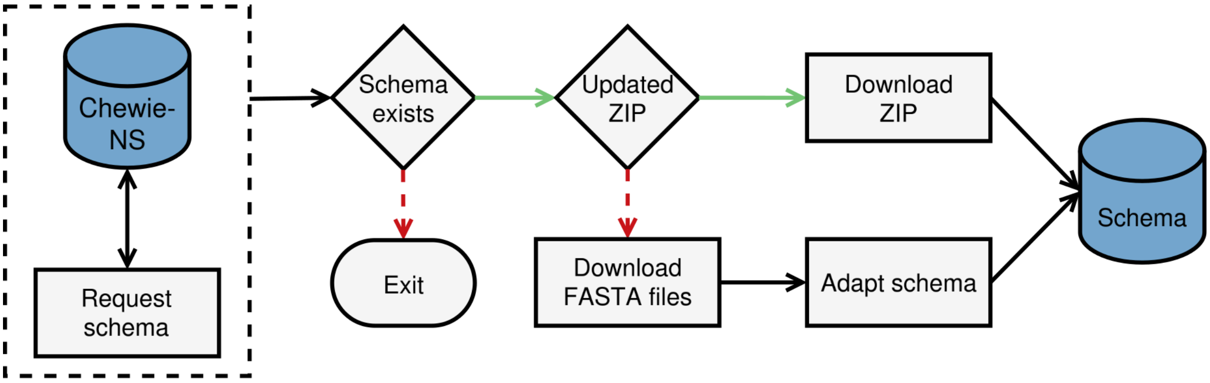

Fig. S9. The *DownloadSchema* module imports schemas from Chewie-NS. The process starts by sending a request with species and schema identifiers to Chewie-NS. If the schema exists, the process checks for a compressed and up-to-date version of the schema to download. If the compressed schema in Chewie-NS is for the latest version of the schema, the compressed schema is downloaded and uncompressed to get a ready-to-use schema. Otherwise, the process will send requests to retrieve the FASTA files with the alleles for all loci and determine the representative alleles with the *PrepExternalSchema* module to create the schema locally. Grey rectangle icons represent analysis steps. Diamond icons represent conditional statements, with green arrows used when the condition is met and red dashed arrows otherwise. The blue cylinder icons represent schemas.

Fig. S10. Diagram of the *LoadSchema* module.

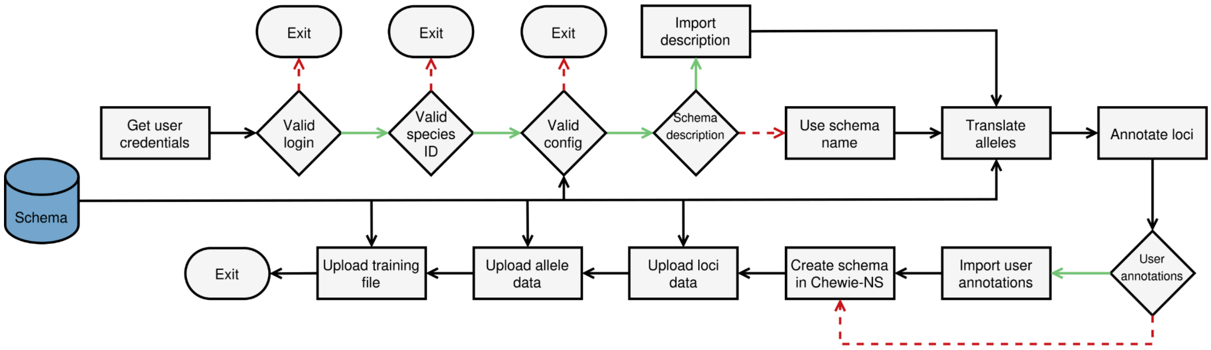

Fig. S10. The *LoadSchema* module uploads local schemas to Chewie-NS. The process starts by requesting the user credentials to ensure that the user has contributor privileges. Only contributors are allowed to upload schemas to Chewie-NS. If the user is a contributor, the process checks if the species identifier provided by the user is valid and if the species is listed in Chewie-NS. After this step, the process reads the schema's configuration file to validate the schema parameter values and ensure that there is only a single value associated with each parameter. The initial validation steps are followed by the upload of the schema data to Chewie-NS. The process reads the schema description, if the user provided one, or uses the schema name as description. The alleles are translated and annotation terms for the loci are obtained through UniProt's SPARQL endpoint. If the user provides custom loci annotations, the process reads the file provided by the user and adds the custom annotations to the loci annotation data to send to Chewie-NS. After retrieving loci annotations, the process creates the schema in Chewie-NS by sending the schema's parameter values and the list of file hashes to validate schema files uploaded in subsequent steps. The loci are created and linked to the newly created schema by sending the loci identifiers and annotations to Chewie-NS. The loci FASTA files are compressed and uploaded to Chewie-NS to add the allele sequences to the database and link them to the corresponding loci. The last step in the process uploads the training file in the local schema and associates it to the newly created schema in Chewie-NS. After process completion, Chewie-NS will process the data that was sent to make the schema data and statistics available through the website and the API. Grey rectangle icons represent analysis steps. Diamond icons represent conditional statements, with green arrows used when the condition is met and red dashed arrows otherwise. The blue cylinder icon represents a schema.

Fig. S11. Diagram of the *SyncSchema* module.

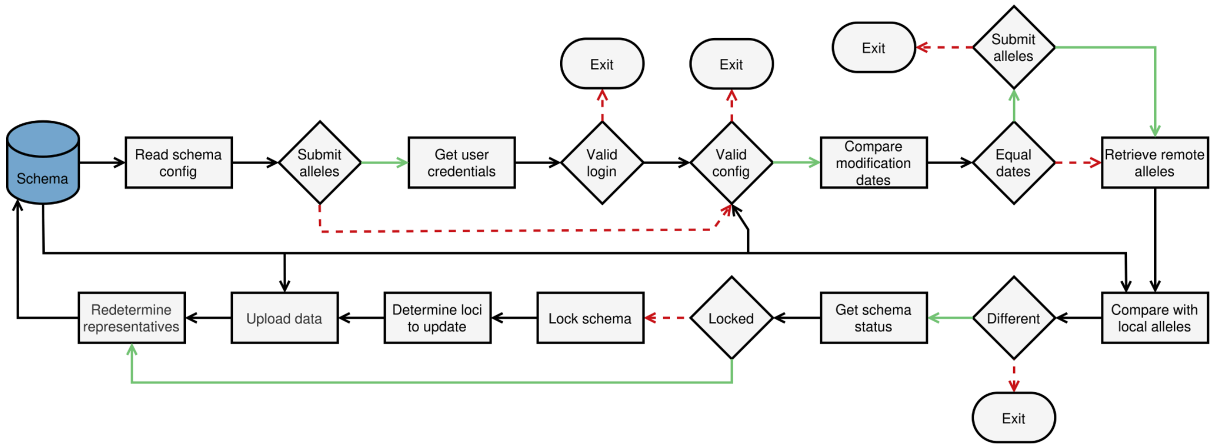

Fig. S11. The *SyncSchema* module retrieves new alleles added to remote schemas in Chewie-NS and submits new alleles added to local schemas to update the remote schemas in Chewie-NS. The process starts by reading the schema's configuration file to get the schema's parameter values and ensure the values match the ones listed in Chewie-NS. If the user wants to submit new alleles identified locally (*--submit*), the process will ask for the user credentials to verify if the user has contributor privileges. Before retrieving or uploading new alleles, the process verifies if the last modification date of the local and remote schemas match. If the dates match and the user does not want to submit new local alleles, the process exits. If the dates do not match or the user wants to submit new local alleles, the process retrieves new alleles added to the remote schema since the last modification date and compares them with the alleles in the local schema. If any alleles are exclusive to the local or remote schema, the process creates updated FASTA files with all the alleles and locks the remote schema to ensure that only the current user can modify the remote schema. The process creates files with the data for the new local alleles and sends them to Chewie-NS, waiting for Chewie-NS to insert the new alleles into the database. After allele insertion in Chewie-NS, the process adapts the updated FASTA files with the *PrepExternalSchema* module to update the local schema and ensure that the local and remote allele identifiers match. If the schema was already locked by another user, the process will skip data upload to Chewie-NS and will update the local schema with new alleles retrieved from Chewie-NS. Grey rectangle icons represent analysis steps. Diamond icons represent conditional statements, with green arrows used when the condition is met and red dashed arrows otherwise. The blue cylinder icon represents a schema.

Fig. S12. Diagram of the *ExtractCgMLST* module.

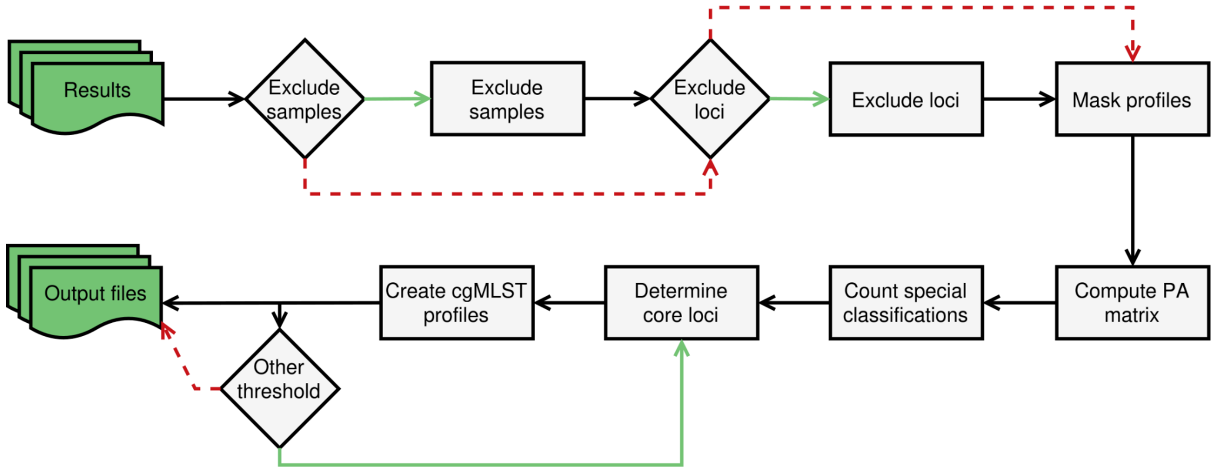

Fig. S12. The *ExtractCgMLST* module determines the set of core loci based on the allelic profiles determined by the *AlleleCall* module. The process starts by excluding loci and samples from the analysis based on lists of loci and samples provided by the user. This allows users to filter out low-quality samples and problematic loci that would affect the determination of the core genome. The filtered allelic profiles are masked to remove the *INF-* prefixes from newly inferred alleles and substitute special classifications by *0*. The masked profiles are used to compute a loci presence-absence matrix and count the number of special classifications per sample. The presence-absence matrix is also used to determine the set of core loci based on the default loci presence thresholds of 0.9, 0.95 and 1, or based on threshold values specified by the user. The process creates output files with the list of loci and allelic profiles per threshold and creates an HTML file with a scatter plot representing the core genome size variation for each threshold. The green document icons represent input and output files. Grey rectangle icons represent analysis steps. Diamond icons represent conditional statements, with green arrows used when the condition is met and red dashed arrows otherwise.

Fig. S13. Runtime and peak memory usage for the four execution modes available in chewBBACA 3.

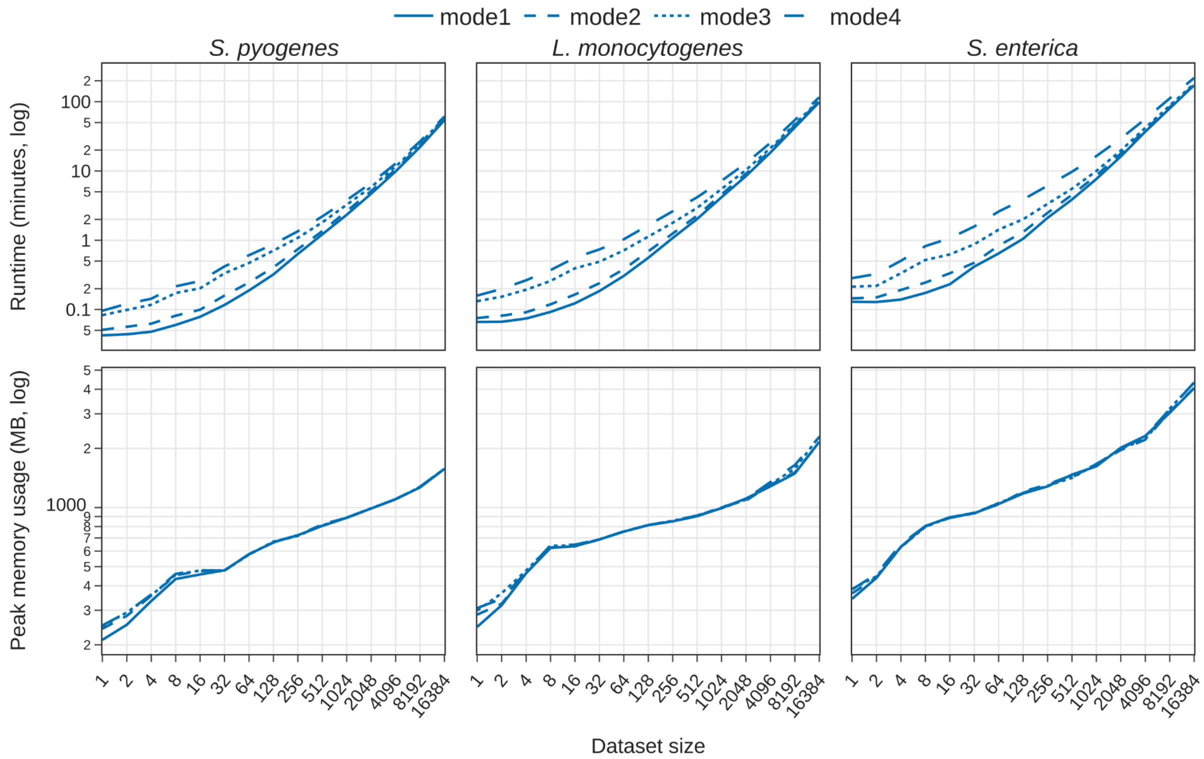

Fig. S13. Runtime and peak memory usage were measured for the allele calling of datasets with 1 to 16384 strains for three bacterial species: *Streptococcus pyogenes*, *Listeria monocytogenes*, and *Salmonella enterica*. The benchmark was performed with five replicates per dataset size, except for the complete dataset (n=16,384 genomes). The values shown are the mean of the replicate values for each dataset. Runtime was measured as the elapsed real time in minutes (logarithmic scale). Peak memory usage was measured as the maximum resident set size in MB (logarithmic scale).

Fig. S14. Pairwise allelic distances differences

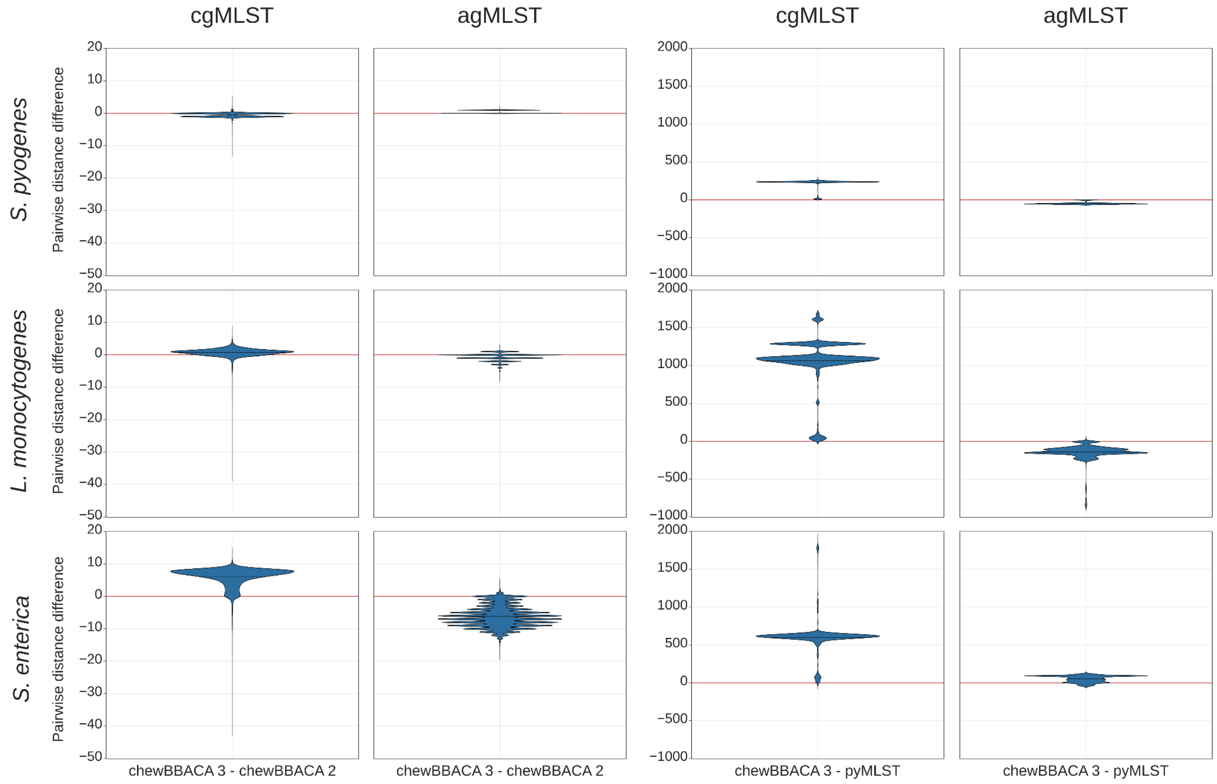

Fig. S14. The pairwise distances differences at the core-genome (cgMLST) and accessory-genome (agMLST) levels were computed by subtracting the allelic distance matrices computed based on chewBBACA 2's and pyMLST's results from the allelic distance matrices computed from chewBBACA 3's results for the complete datasets (n=16,384 genomes). A positive value represents a greater difference with chewBBACA 3 and a negative value a smaller difference with chewBBACA 3 than with the comparator. The zero line in each plot is highlighted in red.

Fig. S15. Proportion of CDSs classified per execution mode for each species' datasets.

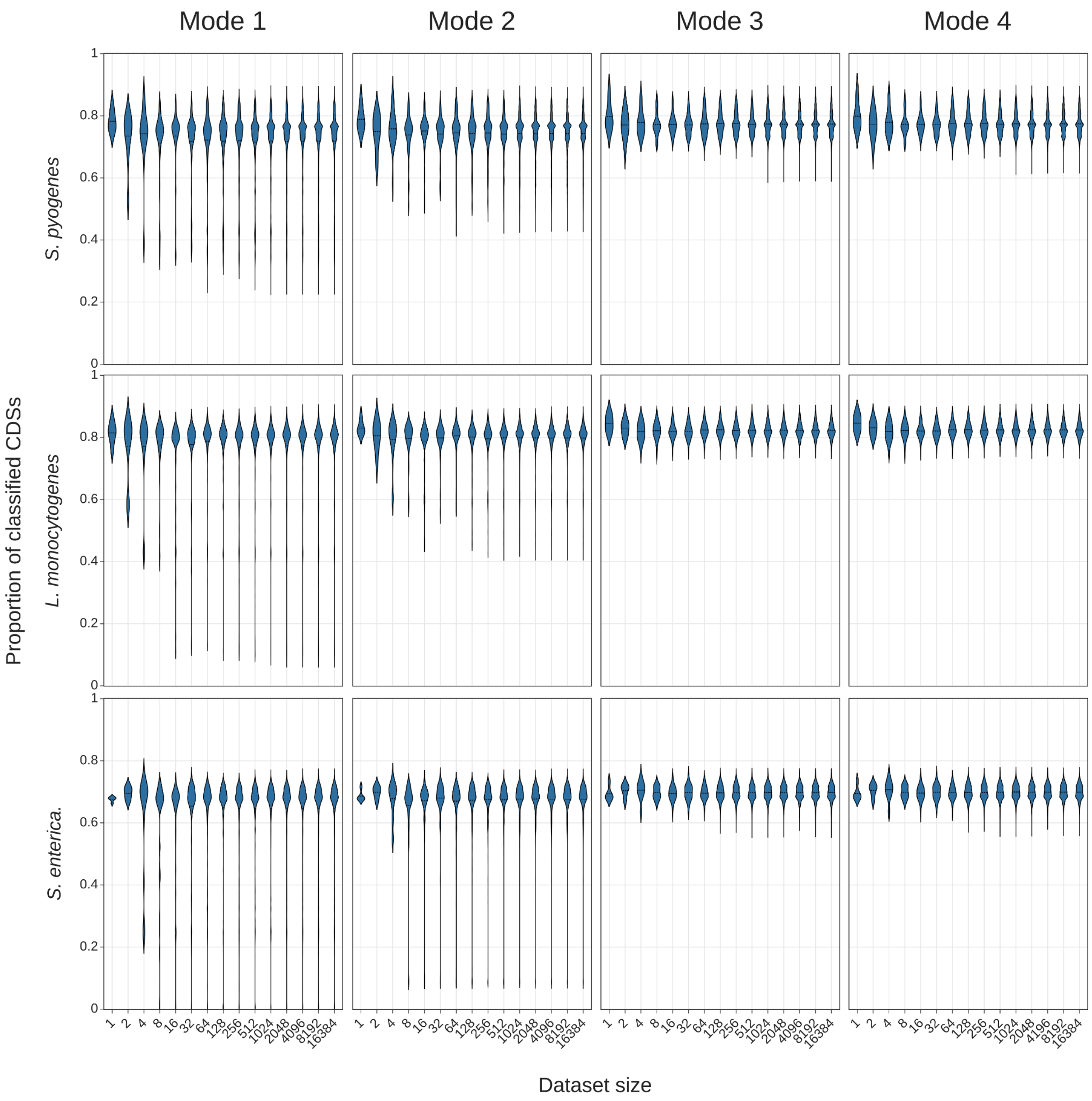

Fig. S15. The proportion of classified CDSs corresponds to the number of CDSs classified by each execution mode divided by the total number of CDSs predicted for each strain by Pyrodigal. The benchmark was performed with five replicates per dataset size, except for the complete dataset (n=16,384 genomes).

Fig. S16. Proportion of schema loci classified per execution mode for each species' datasets.

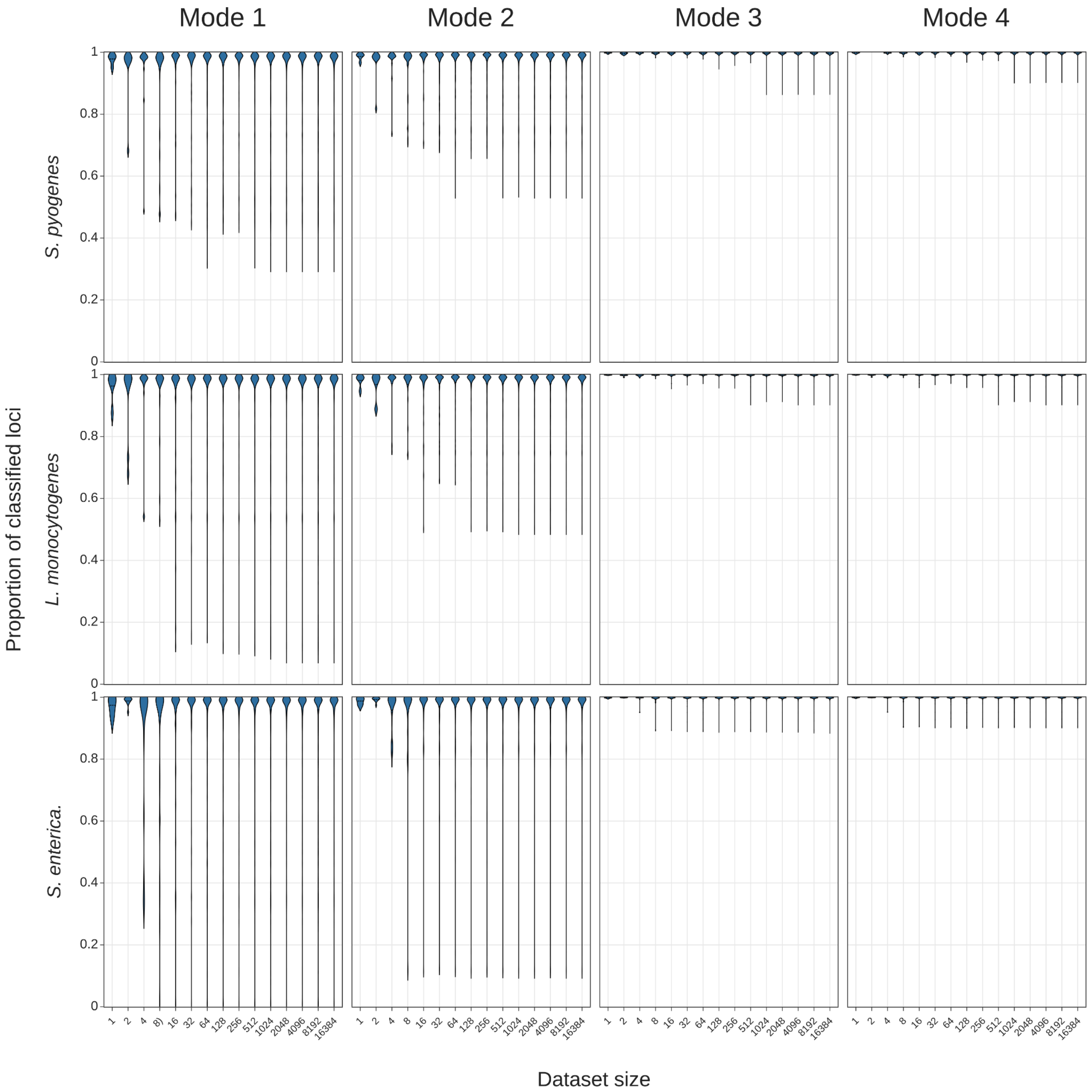

Fig. S16. The proportion of classified loci corresponds to the number of schema loci identified by each execution mode divided by the total number of schema loci. The benchmark was performed with five replicates per dataset size, except for the complete dataset (n=16,384 genomes).

Fig. S17. Classifications counts for the complete dataset (n=16,384 genomes) of *S. pyogenes* per tool.

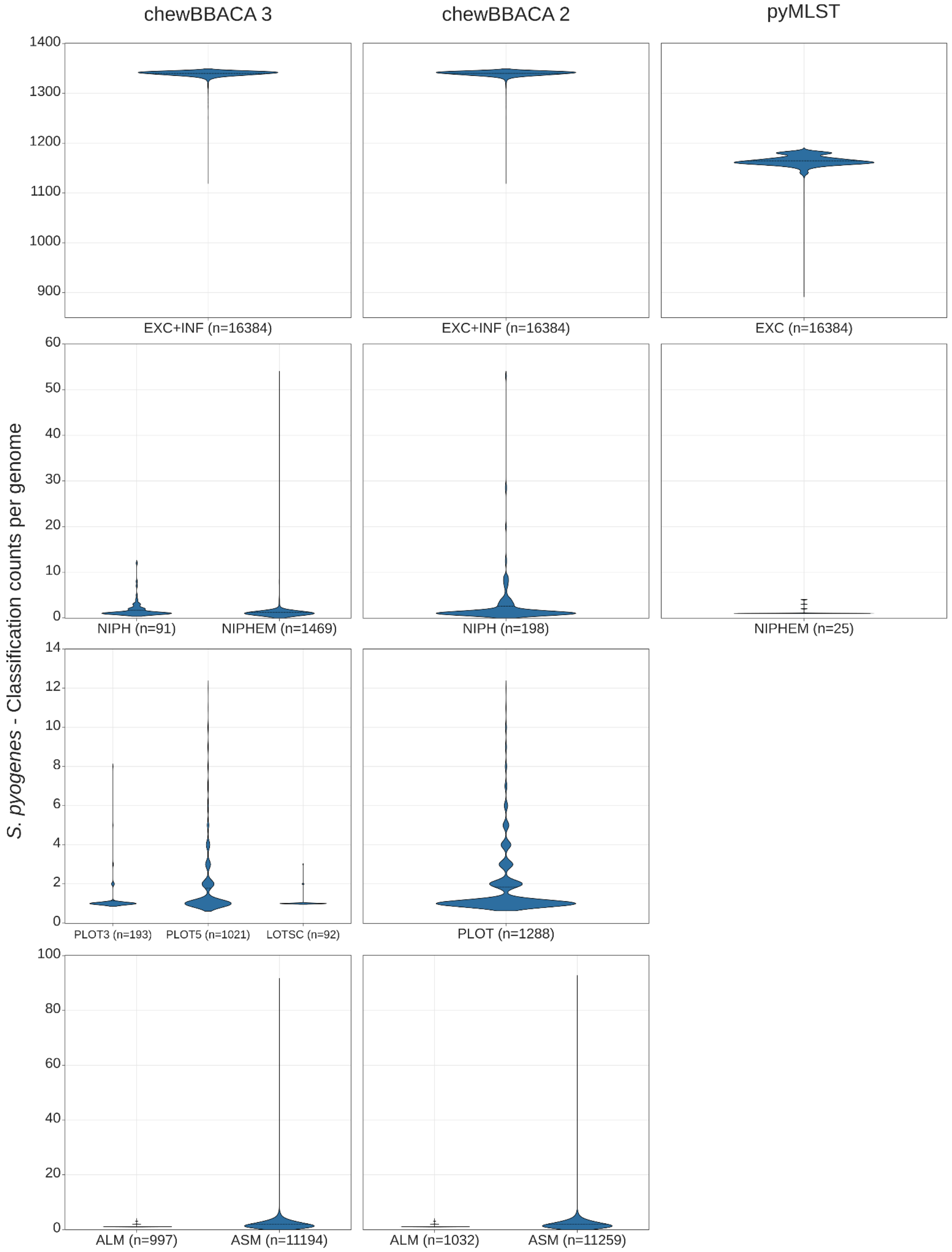

Fig. S17. Each row displays the counts for the special classifications that are equivalent between tools. The x-axis labels show the names of the classifications and the number of genomes with a count above zero inside the parentheses (i.e. genomes with a count of zero for any of the classifications are not included in the plotted values). For pyMLST, the loci with a single matching CDS were converted to EXC and the loci with multiple matches were converted to NIPHEM. The plot is not shown if the tool does not determine a special classification equivalent to the ones displayed in the row.

Fig. S18. Classifications counts for the complete dataset (n=16,384 genomes) of *L. monocytogenes* per tool.

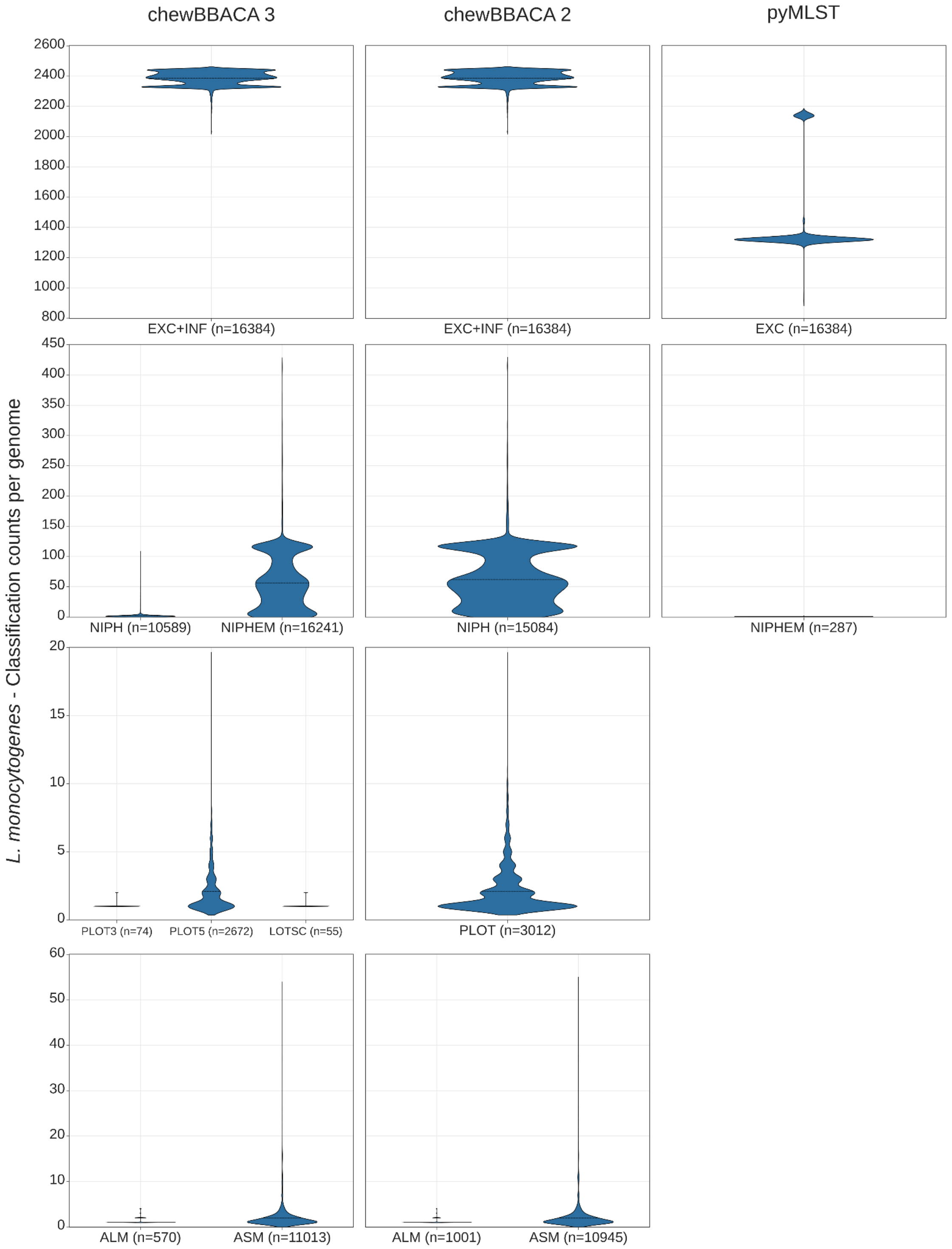

Fig. S18. Each row displays the counts for the special classifications that are equivalent between tools. The x-axis labels show the names of the classifications and the number of genomes with a count above zero inside the parentheses (i.e. genomes with a count of zero for any of the classifications are not included in the plotted values). For pyMLST, the loci with a single matching CDS were converted to EXC and the loci with multiple matches were converted to NIPHEM. The plot is not shown if the tool does not determine a special classification equivalent to the ones displayed in the row.

Fig. S19. Classifications counts for the complete dataset (n=16,384 genomes) of *S. enterica* per tool.

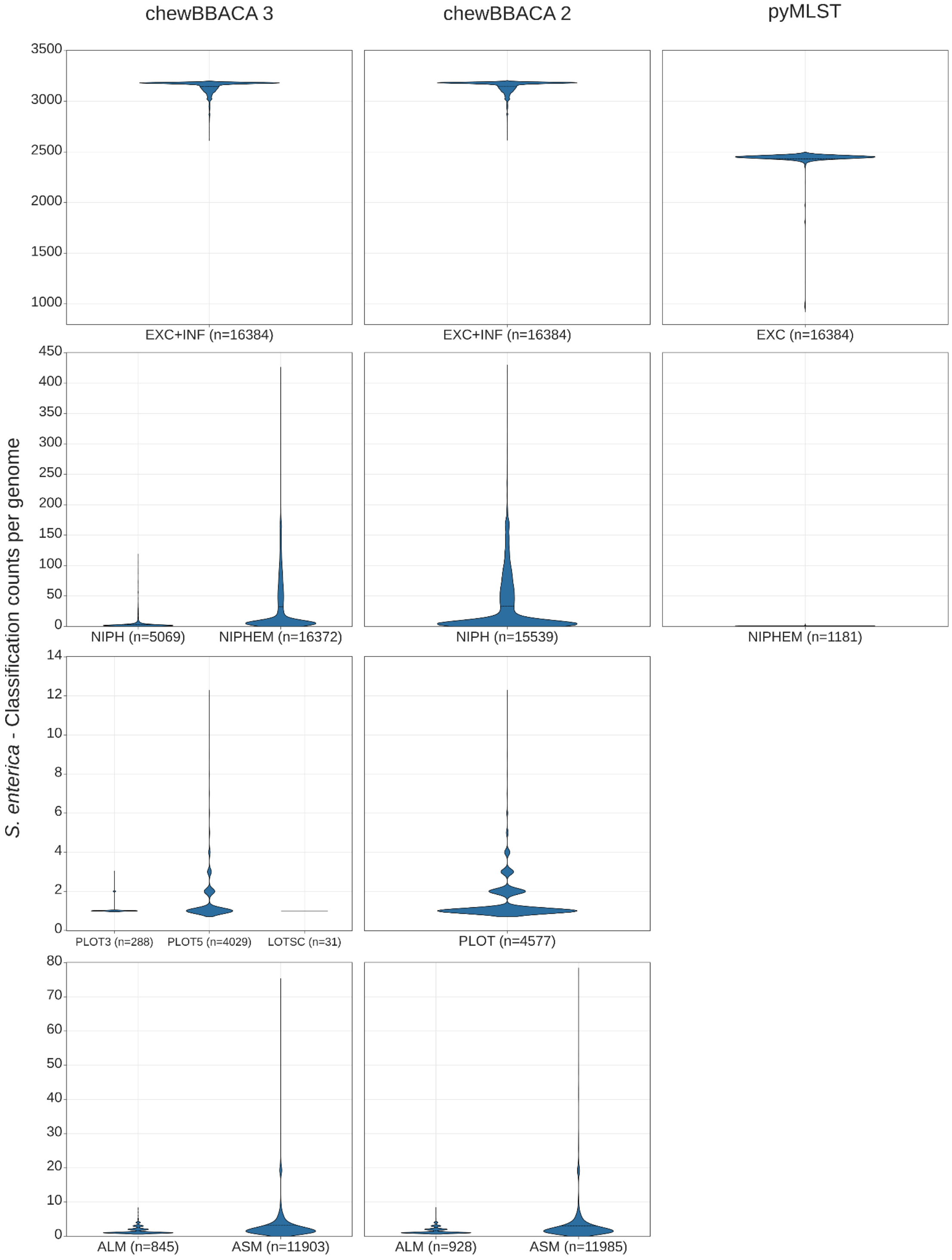

Fig. S19. Each row displays the counts for the special classifications that are equivalent between tools. The x-axis labels show the names of the classifications and the number of genomes with a count above zero inside the parentheses (i.e. genomes with a count of zero for any of the classifications are not included in the plotted values). For pyMLST, the loci with a single matching CDS were converted to EXC and the loci with multiple matches were converted to NIPHEM. The plot is not shown if the tool does not determine a special classification equivalent to the ones displayed in the row.

Fig. S20. Diagram of the *SchemaEvaluator* module.

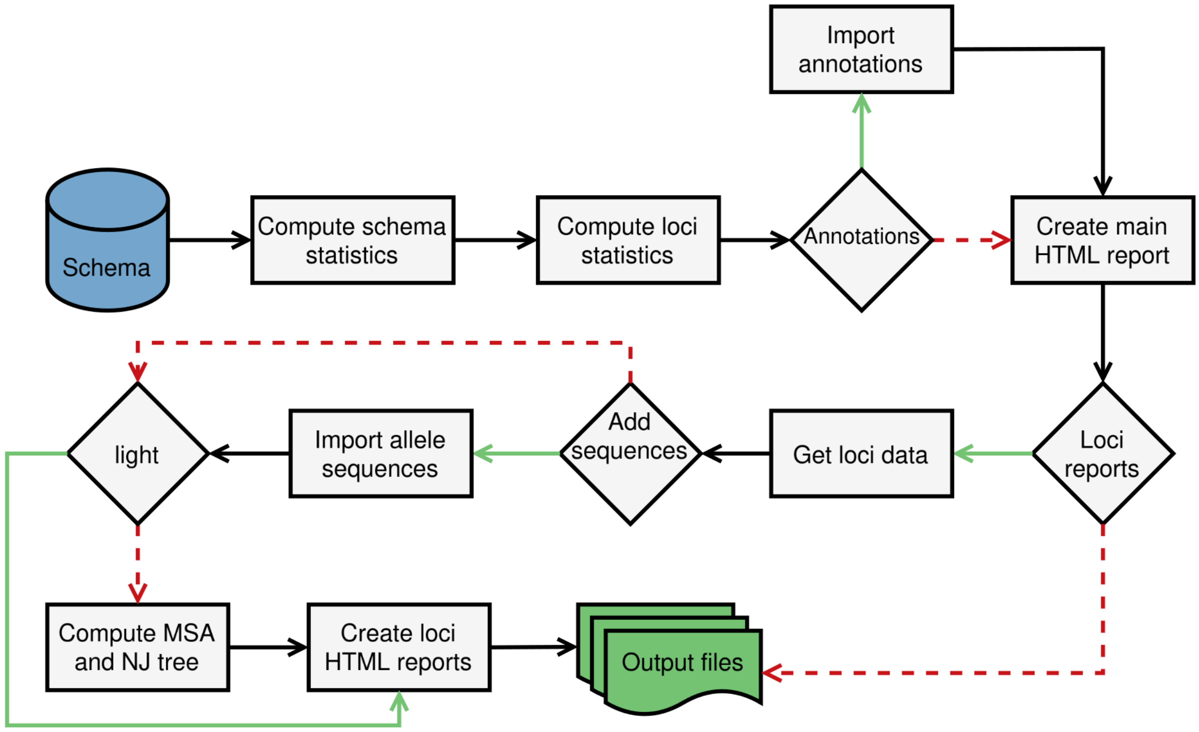

Fig. S20. The *SchemaEvaluator* module analyses a schema to create a report that allows users to explore schema structure and loci diversity interactively. The process starts by computing schema statistics, such as the number of loci and alleles, and loci statistics, such as the number of alleles, allele size statistics, and the number of valid and invalid alleles (e.g. alleles that cannot be translated due to being incomplete, containing ambiguous bases, in-frame stop codons, etc.). The schema and loci statistics are included in interactive data tables and charts on the main page of the HTML report. Loci annotations are imported and included in the main page of the report if provided. If the *--loci-reports* option is provided, the process performs a detailed analysis of each locus to add a separate locus page to the HTML report for each locus. Loci data is analyzed in greater detail to get more detailed statistics per locus. If the *--add-sequences* option is provided, the allele DNA sequences are imported and translated to add DNA and protein sequences to code editors on the locus page, which facilitates identifying and manipulating alleles of interest. Additionally, the process computes a multiple sequence alignment (MSA) for each locus at the protein level with MAFFT to display the MSA and MAFFT's guide tree on interactive components. The MSA and guide tree are not displayed if the *--light* option is provided. The green document icons represent output files. Grey rectangle icons represent analysis steps. Diamond icons represent conditional statements, with green arrows used when the condition is met and red dashed arrows otherwise. The blue cylinder icon represents a schema.

Fig. S21. Diagram of the *UniprotFinder* module.

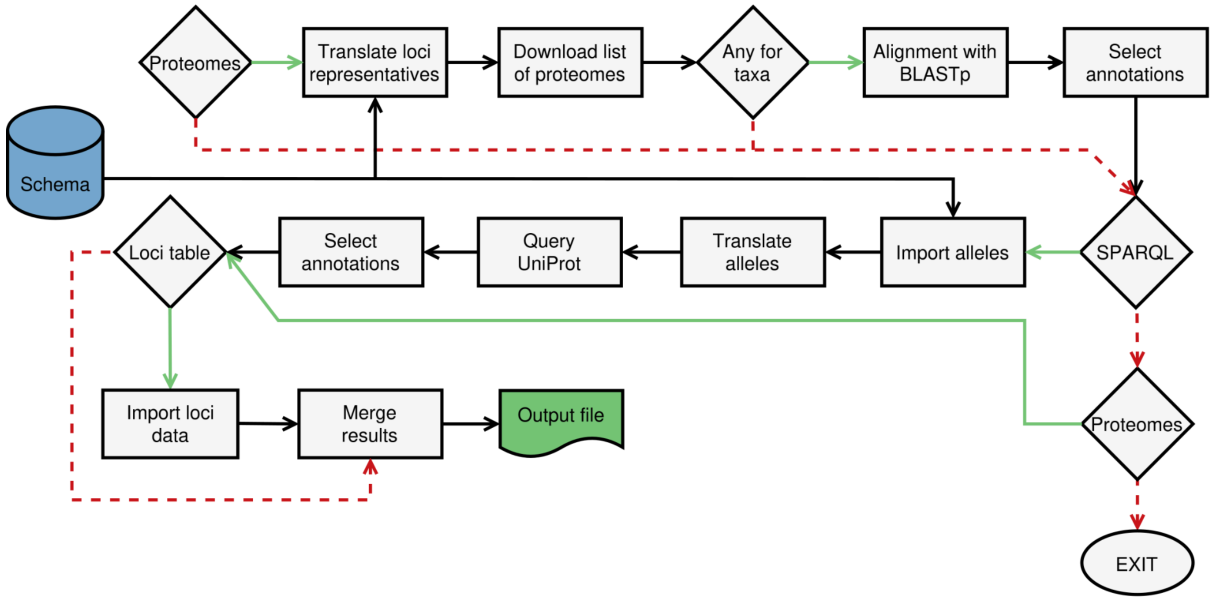

Fig. S21. The *UniprotFinder* module determines annotations for schema loci. The module offers two options to determine annotations: aligning against UniProt's reference proteomes and exact matching through UniProt's SPARQL endpoint. Users must provide at least one valid taxon name to annotate based on the reference proteomes. The process downloads the list of reference proteomes and searches for proteomes for the specified taxa. If there are any proteomes for the specified taxa, they are downloaded, and the loci representative alleles are aligned against the reference proteomes so annotations can be selected based on the BSR. The process searches for annotations through UniProt's SPARQL endpoint by creating queries including the loci alleles and submitting requests to the endpoint. If an allele matches any protein in UniProt, the annotation terms are extracted from the results. The process tries to select the most informative annotation terms. The annotation terms found through both options are merged to create a single annotations table. If the user provides a TSV file with additional loci data, such as the file with CDS coordinates created by the *CreateSchema* and *AlleleCall* modules, the process will add the data in that file to the annotations table. The green document icon represents the output file. Grey rectangle icons represent analysis steps. Diamond icons represent conditional statements, with green arrows used when the condition is met and red dashed arrows otherwise. The blue cylinder icon represents a schema.

Fig. S22. Diagram of the AlleleCallEvaluator module.

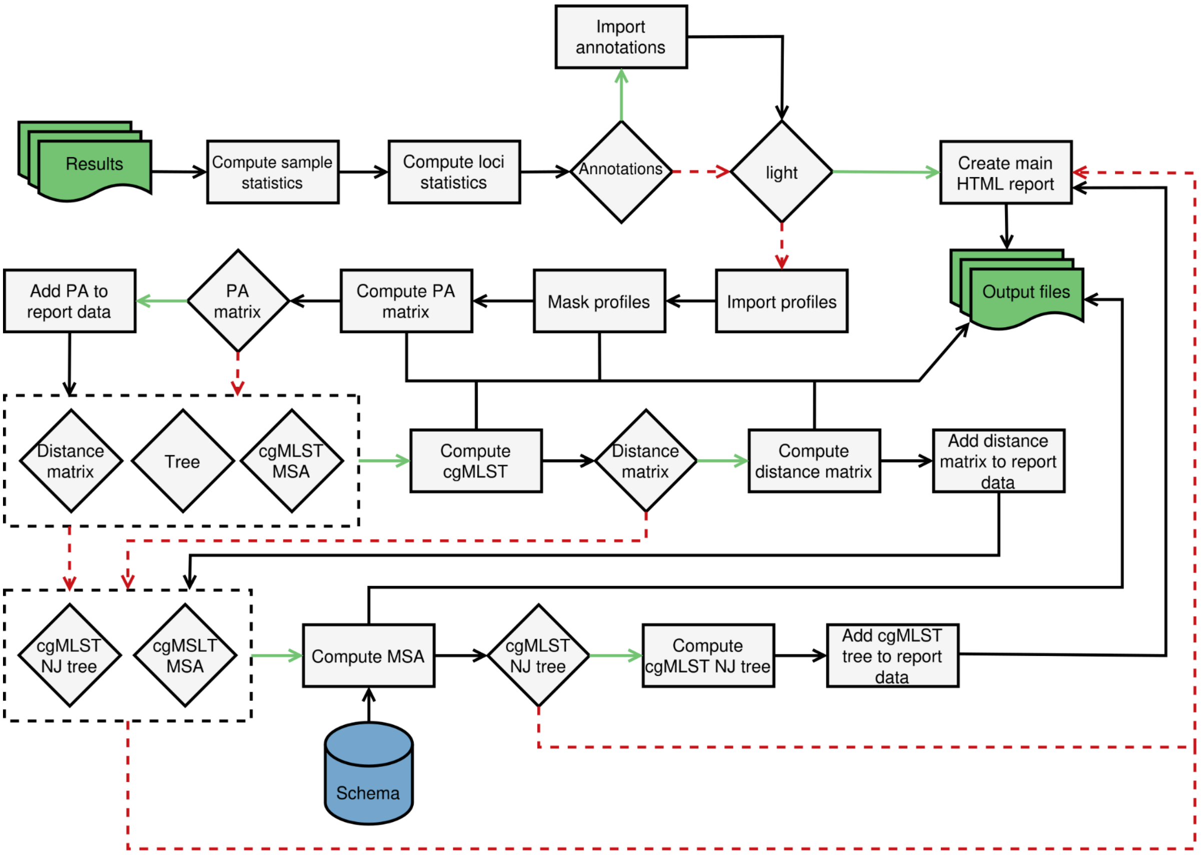

Fig. S22. The *AlleleCallEvaluator* module analyses allele calling results to create a report that allows users to explore results interactively. The process starts by importing and computing sample and loci statistics based on the allele calling results. The sample and loci statistics are included in interactive data tables and charts on the main page of the HTML report. Loci annotations are imported and included in the main page of the report if provided. If the *--light* option is provided, the process does not add more information to the report. Otherwise, the allelic profiles are imported and masked to remove *INF-* prefixes and substitute special classifications by 0. The masked profiles serve as the basis for computing a presence-absence (PA) matrix, enabling the determination of the set of loci that constitute the core genome. The profile data for the core loci are used to compute a matrix of allelic distances. The core loci alleles identified per strain and locus are imported to compute the cgMLST alignment that FastTree uses to compute a Neighbour-Joining (NJ) tree. The PA and distance matrices and NJ tree data are included in the report to be displayed and explored interactively. The green document icons represent input and output files. Grey rectangle icons represent analysis steps. Diamond icons represent conditional statements, with green arrows used when the condition is met and red dashed arrows otherwise. The blue cylinder icon represents a schema.

Fig. S23. Number of additional loci found by chewBBACA 3 for 264 *S. pyogenes* strains.

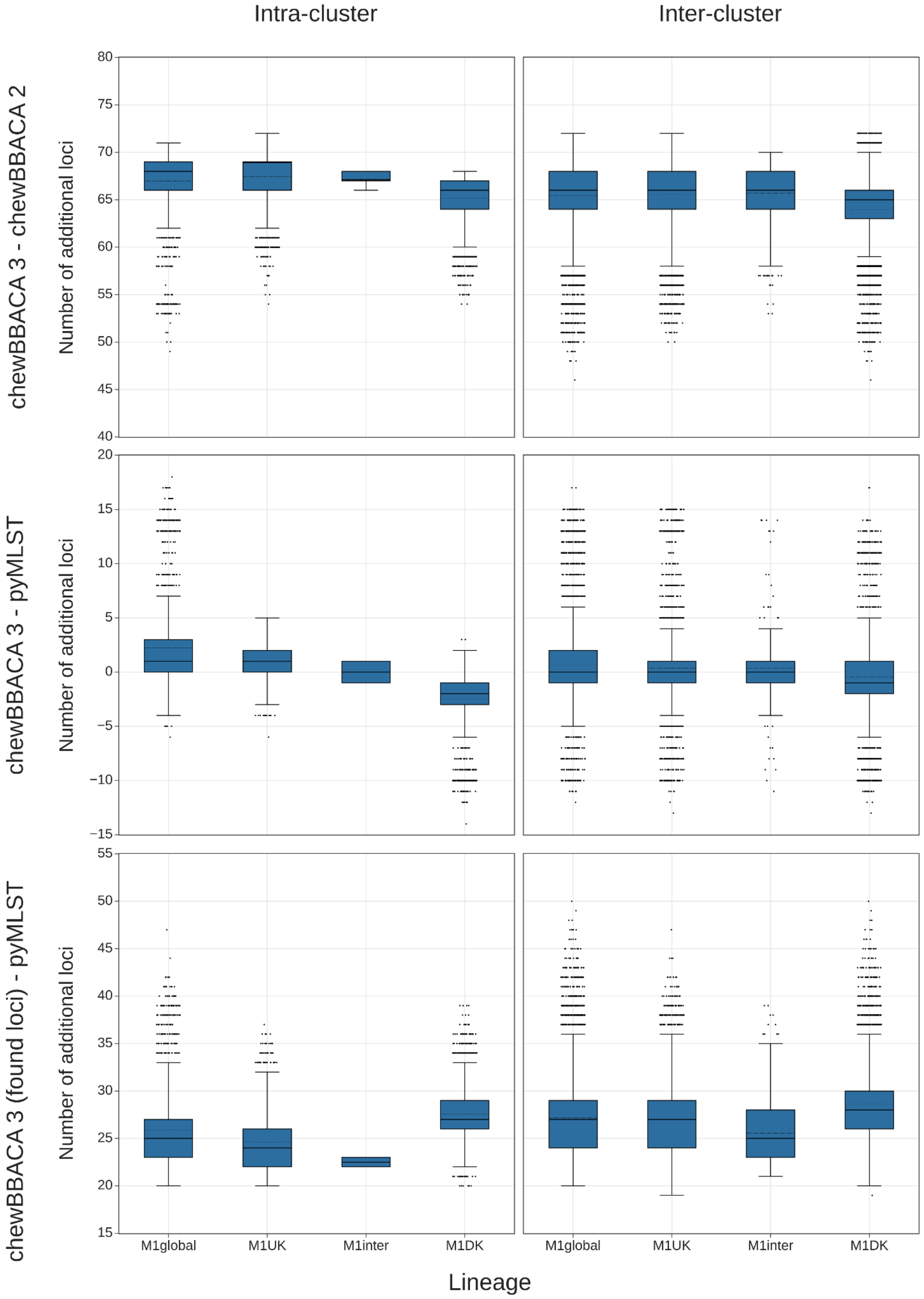

Fig. S23. Number of additional loci for which a valid allele was found for each strain by chewBBACA 3 at the intra- and inter-cluster level for 264 *S. pyogenes* strains grouped by lineage (n=74 $M1_{global}$ strains, n=88 $M1_{UK}$ strains, n=4 $M1_{inter}$ strains, and n=98 $M1_{DK}$ strains). The number of additional loci were computed by subtracting the number of loci for which a valid allele was identified for each strain (exact matches or new alleles) by chewBBACA 2 and pyMLST from the number of valid loci found by chewBBACA 3. Positive values indicate that chewBBACA 3 found more valid loci than chewBBACA 2 and pyMLST. Negative values indicate the contrary. The third row [chewBBACA 3 (found loci) - pyMLST] shows the number of additional valid loci found by chewBBACA 3 compared to pyMLST if the special classifications assigned by chewBBACA 3 are counted as valid loci. This comparison is important because pyMLST will classify some alleles as valid that do not meet the criteria to be considered valid by chewBBACA and this reflects a looser or more stringent criteria of the tools.

Fig. S24. Number of additional loci found by chewBBACA 3 for 501 *S. pneumoniae* strains grouped by Sequence Type.

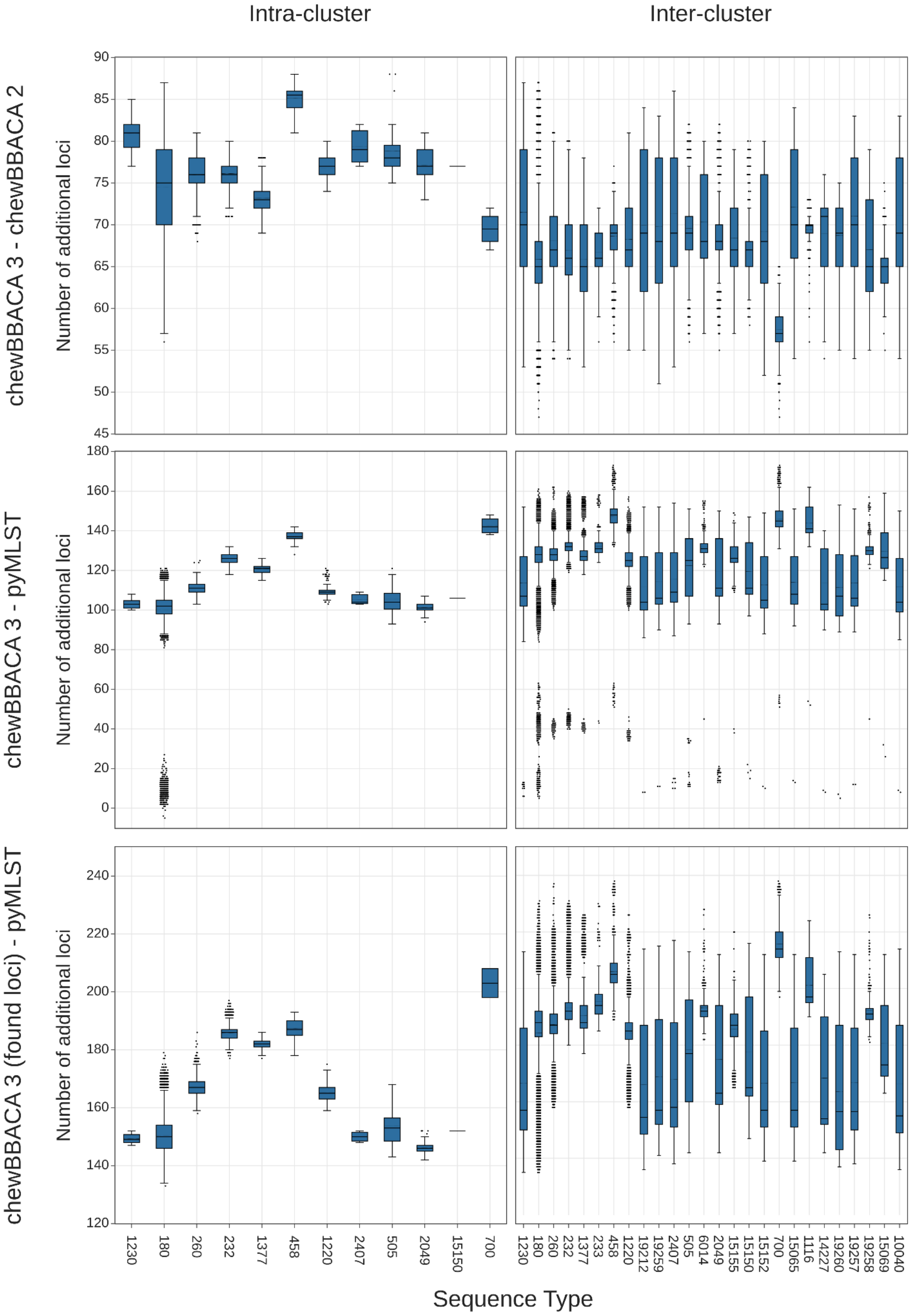

Fig. S24. Number of additional loci for which a valid allele was found for each strain by chewBBACA 3 at the intra- and inter-cluster level for 501 *S. pneumoniae* strains grouped by Sequence Type (ST) (n=6 ST1230 strains, n=249 ST180 strains, n=52 ST 260 strains, n=88 ST232 strains, n=26 ST1377 strains, n=13 ST458 strains, n=21 ST1220 strains, n=3 ST2407 strains, n=9 ST505 strains, n=14 ST2049 strains, n=2 ST15150 strains, n=4 ST700 strains, n=1 strain for each of the remaining STs). The number of additional loci were computed by subtracting the number of loci for which a valid allele was identified for each strain (exact matches or new alleles) by chewBBACA 2 and pyMLST from the number of valid loci found by chewBBACA 3. Positive values indicate that chewBBACA 3 found more valid loci than chewBBACA 2 and pyMLST. Negative values indicate the contrary. The third row [chewBBACA 3 (found loci) - pyMLST] shows the number of additional valid loci found by chewBBACA 3 compared to pyMLST if the special classifications assigned by chewBBACA 3 are counted as valid loci. This comparison is important because pyMLST will classify some alleles as valid that do not meet the criteria to be considered valid by chewBBACA and this reflects a looser or more stringent criteria of the tools.

Fig. S25. Number of additional loci found by chewBBACA 3 for 501 *S. pneumoniae* strains grouped by Global Pneumococcal Sequence Cluster.

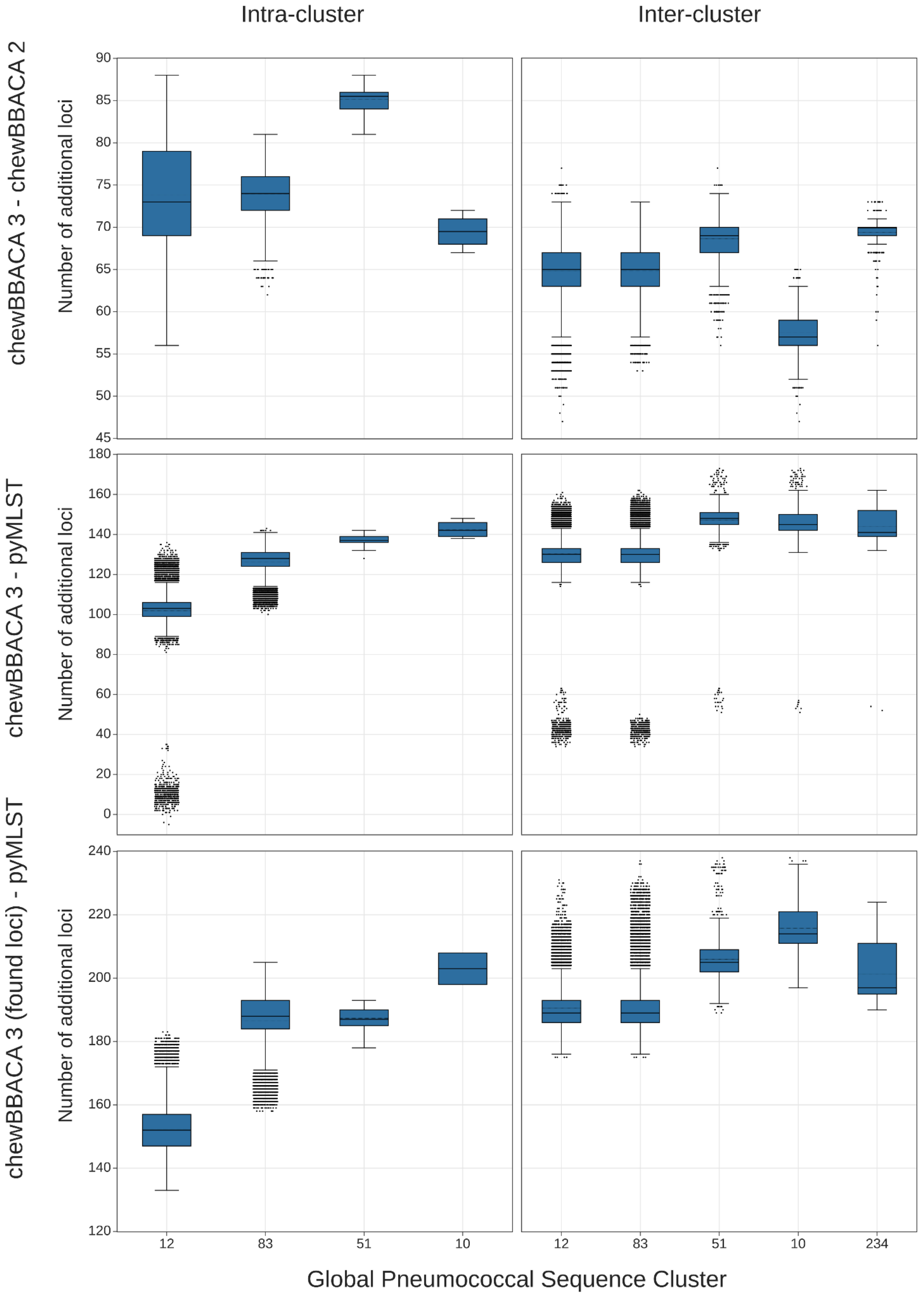

Fig. S25. Number of additional loci found for each strain by chewBBACA 3 at the intra- and inter-cluster level for 501 *S. pneumoniae* strains grouped by Global Pneumococcal Sequence Cluster (GPSC) (n=281 GPSC12 strains, n=185 GPSC83 strains, n=13 GPSC51 strains, n=4 GPSC10 strains, and n=1 GPSC234 strain). The number of additional loci were computed by subtracting the number of loci for which a valid allele was identified for each strain (exact matches or new alleles) by chewBBACA 2 and pyMLST from the number of valid loci found by chewBBACA 3. Positive values indicate that chewBBACA 3 found more valid loci than chewBBACA 2 and pyMLST. Negative values indicate the contrary. The third row [chewBBACA 3 (found loci) - pyMLST] shows the number of additional valid loci found by chewBBACA 3 compared to pyMLST if the special classifications assigned by chewBBACA 3 are counted as valid loci. This comparison is important because pyMLST will classify some alleles as valid that do not meet the criteria to be considered valid by chewBBACA and this reflects a looser or more stringent criteria of the tools.

Fig. S26. Number of additional loci found by chewBBACA 3 for 501 *S. pneumoniae* strains grouped by Azarian et al. clade.

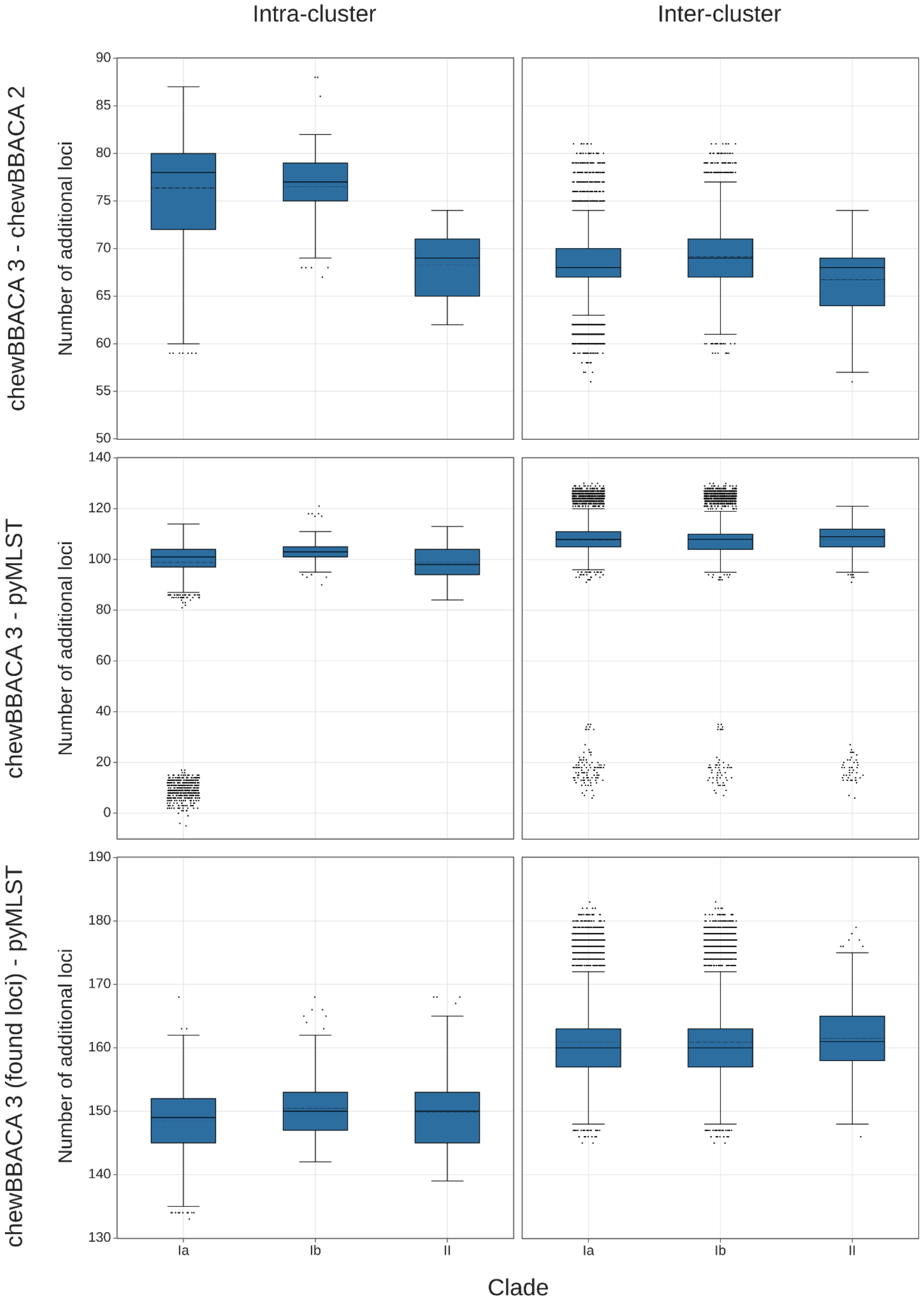

Fig. S26. Number of additional loci found for each strain by chewBBACA 3 at the intra- and inter-cluster level for 501 *S. pneumoniae* strains grouped by Azarian et al. clade (n=241 clade Ia strains, n=28 clade Ib strains, and n=22 clade II strains). The number of additional loci were computed by subtracting the number of loci for which a valid allele was identified for each strain (exact matches or new alleles) by chewBBACA 2 and pyMLST from the number of valid loci found by chewBBACA 3. Positive values indicate that chewBBACA 3 found more valid loci than chewBBACA 2 and pyMLST. Negative values indicate the contrary. The third row [chewBBACA 3 (found loci) - pyMLST] shows the number of additional valid loci found by chewBBACA 3 compared to pyMLST if the special classifications assigned by chewBBACA 3 are counted as valid loci. This comparison is important because pyMLST will classify some alleles as valid that do not meet the criteria to be considered valid by chewBBACA and this reflects a looser or more stringent criteria of the tools.

Fig. S27. Number of additional loci found by chewBBACA 3 for 501 *S. pneumoniae* strains grouped by Kwun et al. clade.

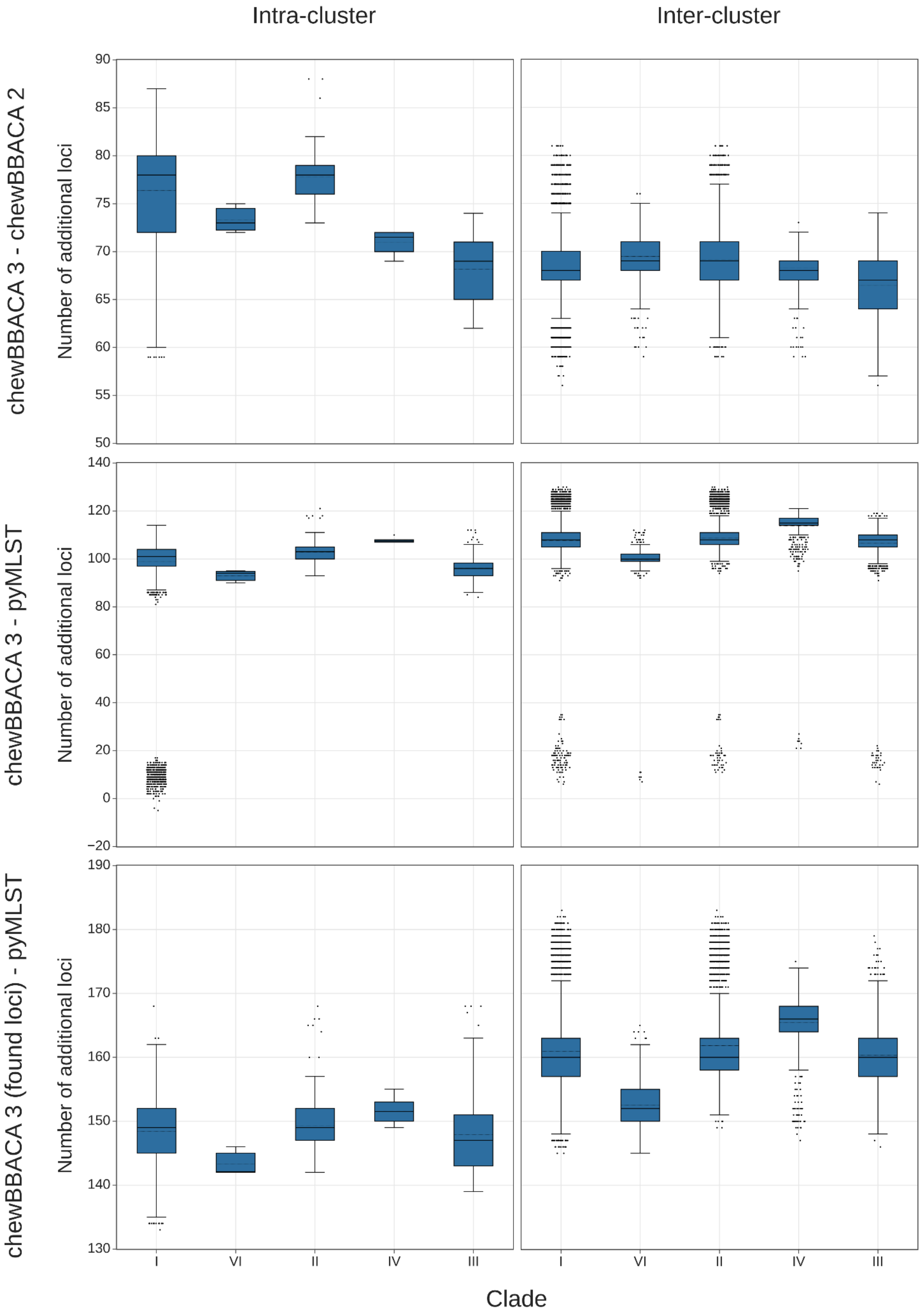

Fig. S27. Number of additional loci found for each strain by chewBBACA 3 at the intra- and inter-cluster level for 501 *S. pneumoniae* strains grouped by Kwun et al. clade (n=241 clade I strains, n=25 clade II strains, n=18 clade III strains, n=4 clade IV strains, n=3 clade VI strains). The number of additional loci were computed by subtracting the number of loci for which a valid allele was identified for each strain (exact matches or new alleles) by chewBBACA 2 and pyMLST from the number of valid loci found by chewBBACA 3. Positive values indicate that chewBBACA 3 found more valid loci than chewBBACA 2 and pyMLST. Negative values indicate the contrary. The third row [chewBBACA 3 (found loci) - pyMLST] shows the number of additional valid loci found by chewBBACA 3 compared to pyMLST if the special classifications assigned by chewBBACA 3 are counted as valid loci. This comparison is important because pyMLST will classify some alleles as valid that do not meet the criteria to be considered valid by chewBBACA and this reflects a looser or more stringent criteria of the tools.

Fig. S28. Intra- and inter-cluster distance differences for 264 *S. pyogenes* strains.

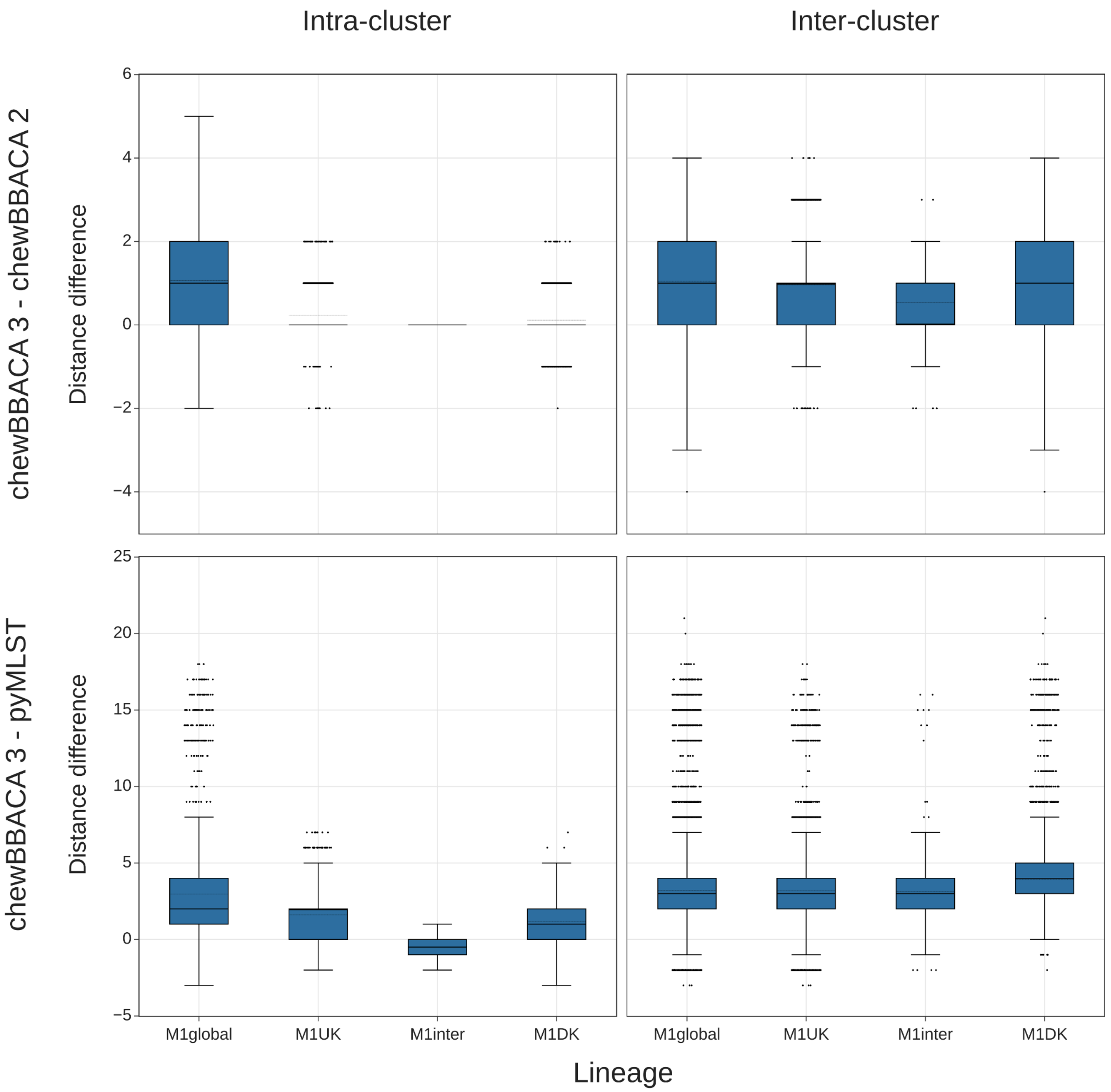

Fig. S28. Intra- and inter-cluster pairwise distances for 264 *S. pyogenes* strains grouped by lineage (n=74 $M1_{global}$ strains, n=88 $M1_{UK}$ strains, n=4 $M1_{inter}$ strains, and n=98 $M1_{DK}$ strains). The intra- and inter-cluster pairwise distance differences were computed by subtracting the allelic pairwise distances computed based on chewBBACA 2's and pyMLST's allele calling results from the pairwise allelic distances computed based on chewBBACA 3's allele calling results. Positive values indicate that the pairwise distances computed based on chewBBACA 3's results are greater than the ones computed from chewBBACA 2's and pyMLST's results. Negative values indicate the contrary.

Fig. S29. Intra- and inter-cluster distance differences for 501 *S. pneumoniae* strains grouped by sequence type.

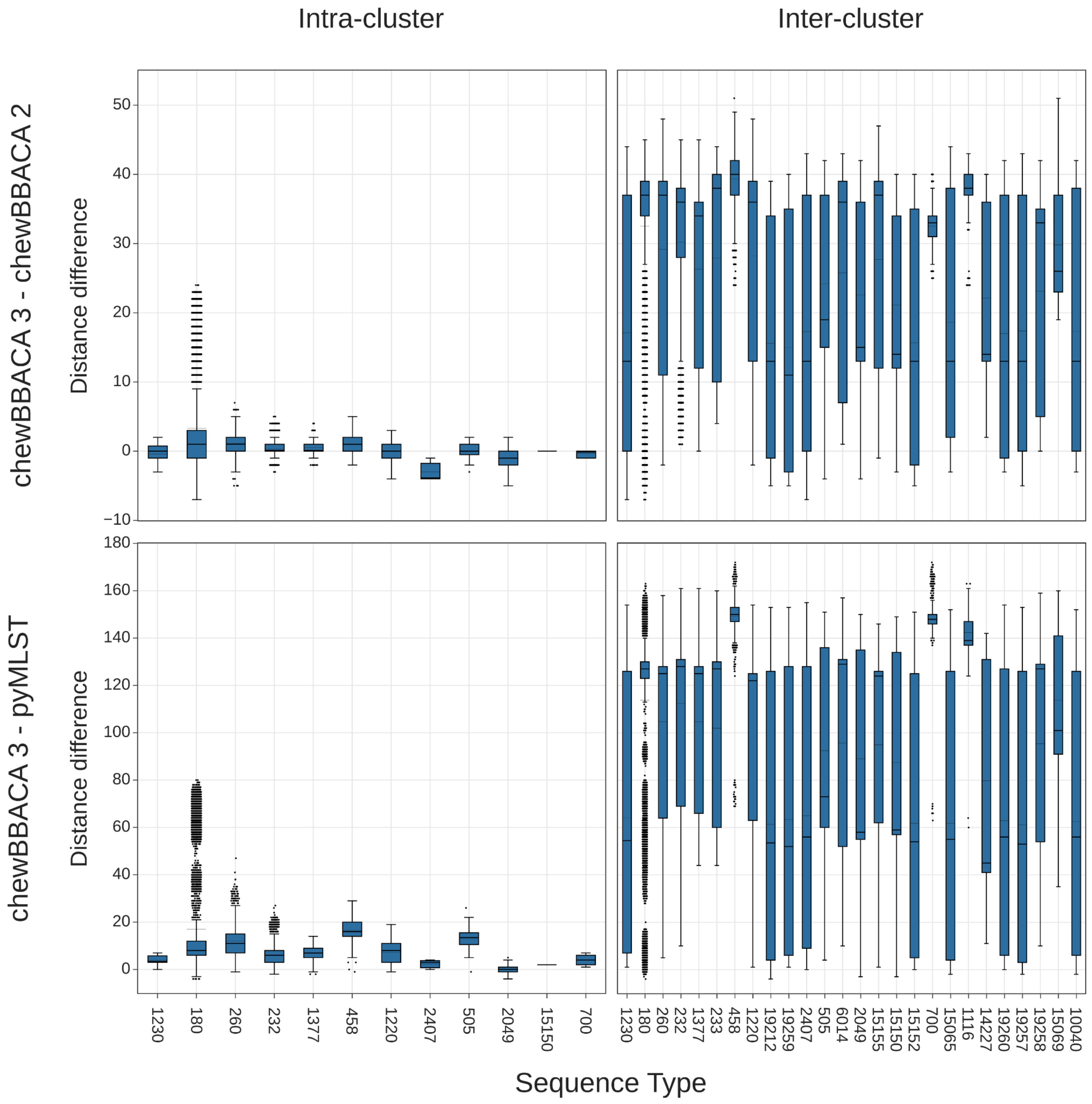

Fig. S29. Intra- and inter-cluster pairwise distances for 501 *S. pneumoniae* strains grouped by Sequence Type (ST) (n=6 ST1230 strains, n=249 ST180 strains, n=52 ST 260 strains, n=88 ST232 strains, n=26 ST1377 strains, n=13 ST458 strains, n=21 ST1220 strains, n=3 ST2407 strains, n=9 ST505 strains, n=14 ST2049 strains, n=2 ST15150 strains, n=4 ST700 strains, n=1 strain for each of the remaining STs). The intra- and inter-cluster pairwise distance differences were computed by subtracting the pairwise allelic distances computed based on chewBBACA 2's and pyMLST's allelic profiles from the pairwise allelic distances computed based on chewBBACA 3's allelic profiles. Positive values indicate that the pairwise distances computed based on chewBBACA 3's results are greater than the ones computed from chewBBACA 2's and pyMLST's results. Negative values indicate the contrary. For the intra-cluster distance differences, no values are shown for STs with a single strain.

Fig. S30. Intra- and inter-cluster distance differences for 501 *S. pneumoniae* strains grouped by Global Pneumococcal Sequence Cluster.

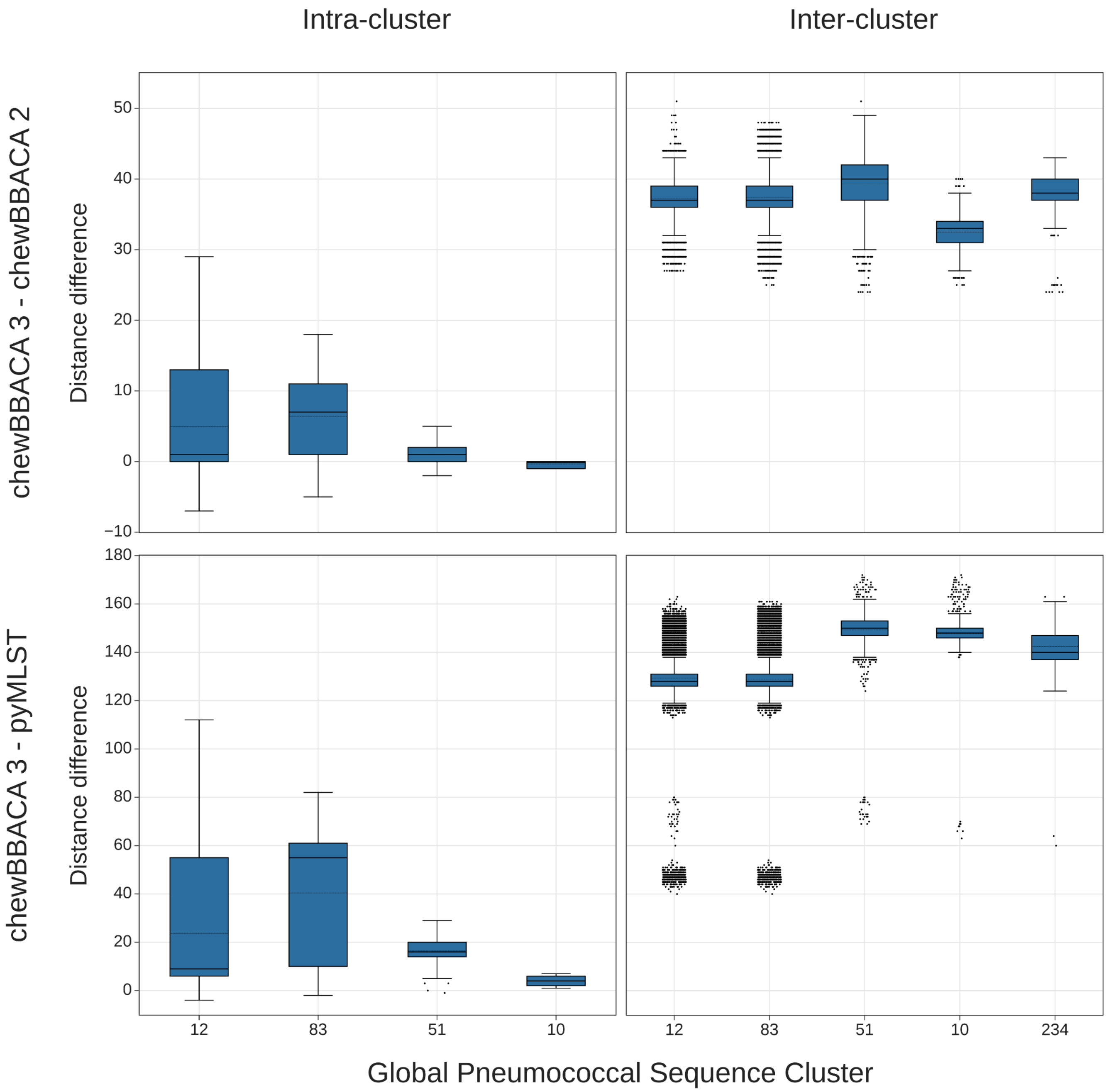

Fig. S30. Intra- and inter-cluster pairwise distances for 501 *S. pneumoniae* strains grouped by Global Pneumococcal Sequence Cluster (GPSC) (n=281 GPSC12 strains, n=185 GPSC83 strains, n=13 GPSC51 strains, n=4 GPSC10 strains, and n=1 GPSC234 strain). The intra- and inter-cluster pairwise distance differences were computed by subtracting the pairwise allelic distances computed based on chewBBACA 2's and pyMLST's allelic profiles from the pairwise allelic distances computed based on chewBBACA 3's allelic profiles. Positive values indicate that the pairwise distances computed based on chewBBACA 3's results are greater than the ones computed from chewBBACA 2's and pyMLST's results. Negative values indicate the contrary. For the intra-cluster distance differences, no values are shown for GPSCs with a single strain.

Fig. S31. Intra- and inter-cluster distance differences for 501 *S. pneumoniae* strains grouped by Azarian et al. clade.

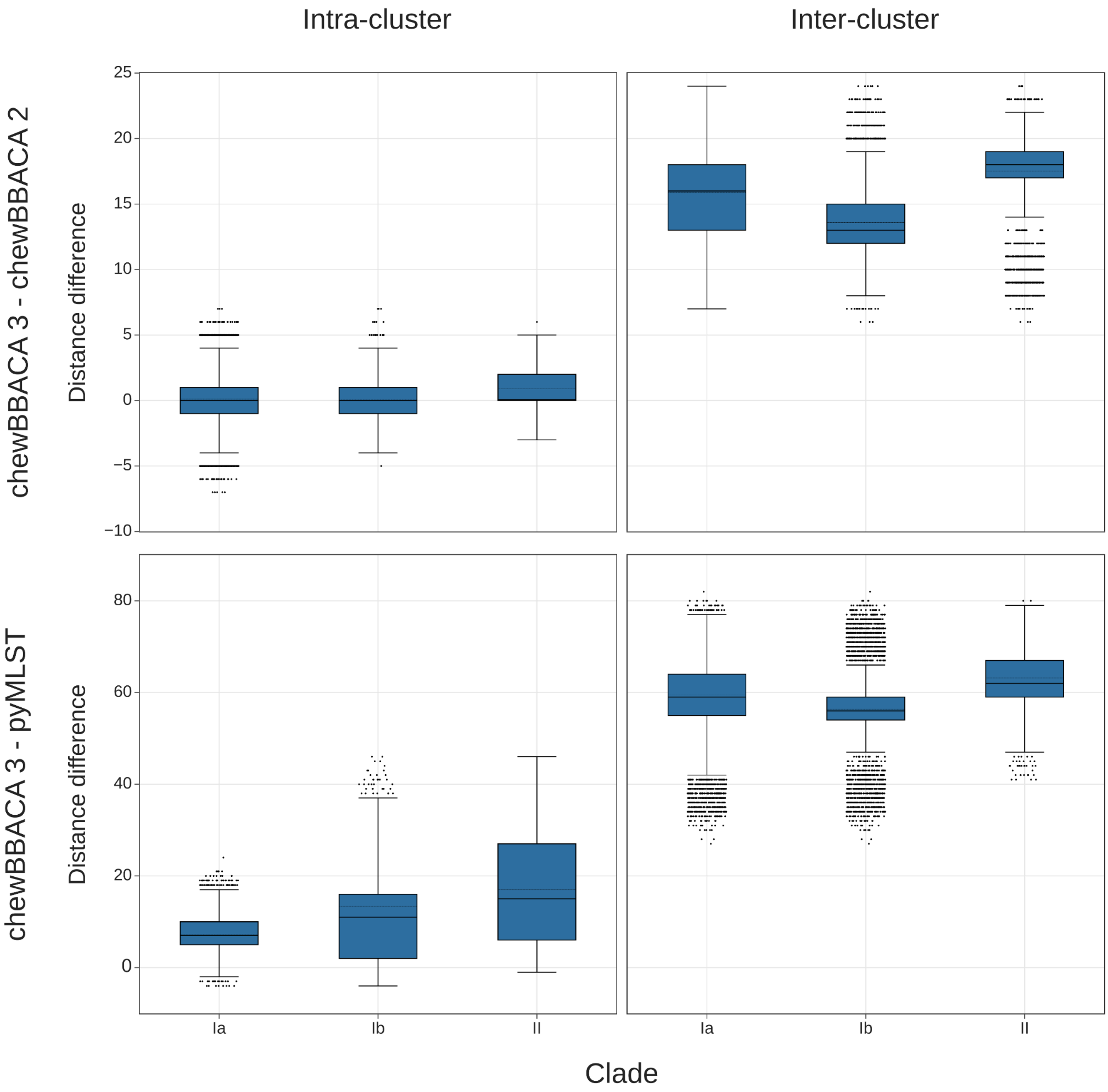

Fig. S31. Intra- and inter-cluster pairwise distances for 501 *S. pneumoniae* strains grouped by Azarian et al. clade (n=241 clade Ia strains, n=28 clade Ib strains, and n=22 clade II strains). The intra- and inter-cluster pairwise distance differences were computed by subtracting the pairwise allelic distances computed based on chewBBACA 2's and pyMLST's allelic profiles from the pairwise allelic distances computed based on chewBBACA 3's allelic profiles. Positive values indicate that the pairwise distances computed based on chewBBACA 3's results are greater than the ones computed from chewBBACA 2's and pyMLST's results. Negative values indicate the contrary.

Fig. S32. Intra- and inter-cluster distance differences for 501 *S. pneumoniae* strains grouped by Kwun et al. clade.

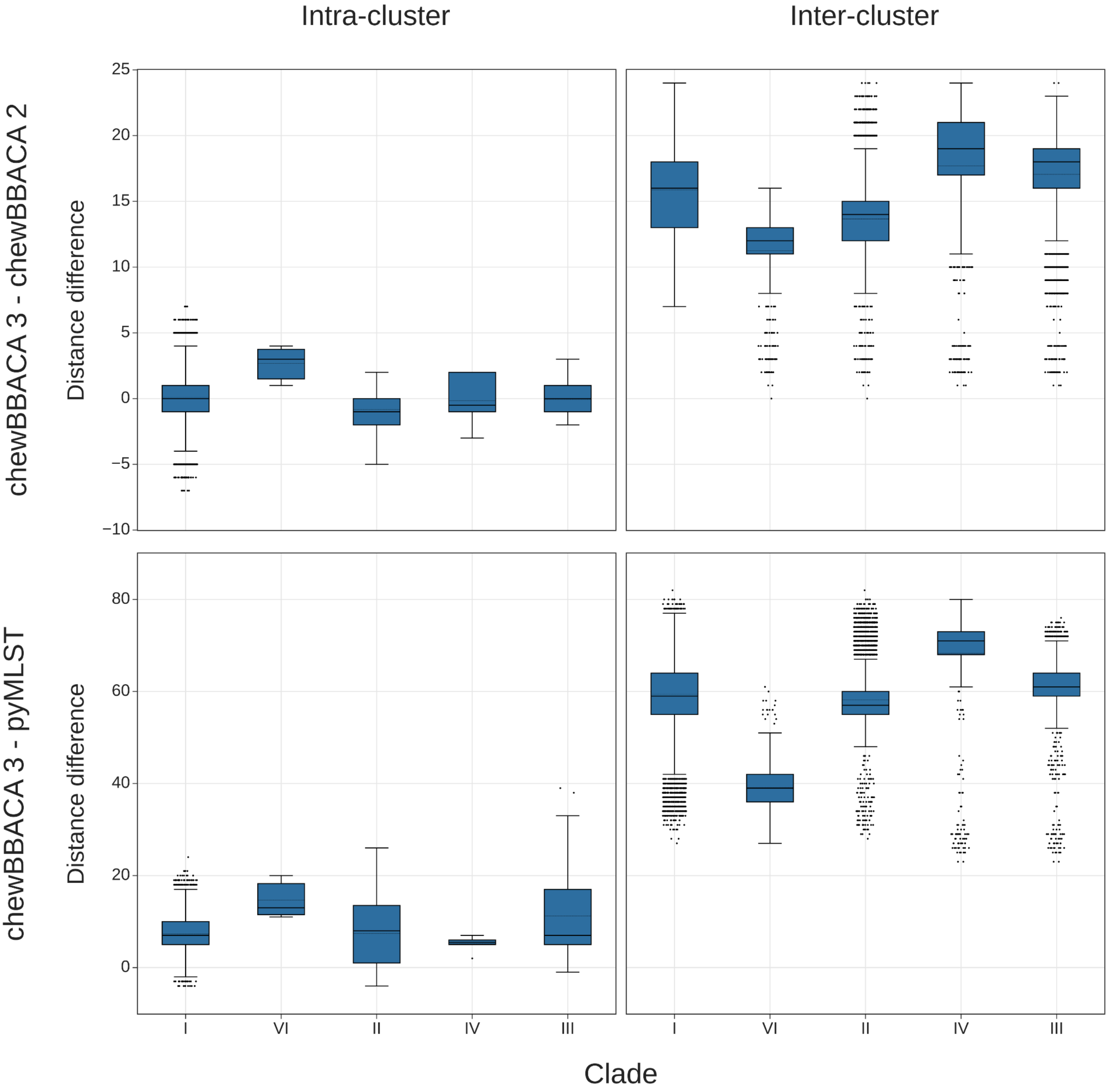

Fig. S32. Intra- and inter-cluster pairwise distances for 501 *S. pneumoniae* strains grouped by Kwun et al. clade (n=241 clade I strains, n=25 clade II strains, n=18 clade III strains, n=4 clade IV strains, n=3 clade VI strains). The intra- and inter-cluster pairwise distance differences were computed by subtracting the pairwise allelic distances computed based on chewBBACA 2's and pyMLST's allelic profiles from the pairwise allelic distances computed based on chewBBACA 3's allelic profiles. Positive values indicate that the pairwise distances computed based on chewBBACA 3's results are greater than the ones computed from chewBBACA 2's and pyMLST's results. Negative values indicate the contrary.
